# SIGEL: a context-aware genomic representation learning framework for spatial genomics analysis

**DOI:** 10.1186/s13059-025-03748-7

**Published:** 2025-09-22

**Authors:** Wenlin Li, Maocheng Zhu, Yucheng Xu, Mengqian Huang, Ziyi Wang, Jing Chen, Hao Wu, Xiaobo Sun

**Affiliations:** 1 School of Data Science, The Chinese University of Hong Kong, Shenzhen, 518172 China; 2 School of Statistics and Mathematics, Zhongnan University of Economics and Law, Wuhan, 430073 China; 3 School of Statistics and Data Science, Nankai University, Tianjin, 300071 China; 4 Faculty of Computer Science and Control Engineering, Shenzhen University of Advanced Technology, Shenzhen, 518055 China; 5 Shenzhen Institute of Advanced Technology, Chinese Academy of Sciences, Shenzhen, 518055 China; 6 Department of Human Genetics, School of Medicine, Atlanta, GA 30322 USA

## Abstract

Spatial transcriptomics (ST) integrates spatial information into genomics, yet methods for generating spatially-informed gene representations are limited and computationally intensive. We present SIGEL, a cost-effective framework that derives gene manifolds from ST data by exploiting spatial genomic context. The resulting SIGEL-generated gene representations (SGRs) are context-aware, biologically meaningful, and robust across samples, making them highly effective for key downstream tasks, including imputing missing genes, detecting spatial expression patterns, identifying disease-related genes and interactions, and improving spatial clustering. Extensive experiments across diverse ST datasets validate SIGEL’s effectiveness and highlight its potential in advancing spatial genomics research.

**Supplementary Information:**

The online version contains supplementary material available at 10.1186/s13059-025-03748-7.

## Background

Distributed gene representations capture the multifaceted nature of genes within high-dimensional spaces, offering profound and quantifiable insights into the intricate mechanisms of gene expression, regulation, and interaction. They pave the way for leveraging machine learning techniques in advancing biomedical research [1], disease diagnosis [2], and the discovery of therapeutic targets [3] with unprecedented precision and efficiency.

Spatial transcriptomics (ST), including high-resolution in situ hybridization-based (e.g., SeqFISH [4]) and high-throughput in situ capturing-based (e.g., 10x Visium [5]) technologies, enables the profiling of spatial gene expression in heterogeneous tissues, providing unprecedented opportunities to characterize spatial distribution of cell types [6], delineate spatial tissue organization [7], gene-gene interactions [8], etc. ST also can reveal spatial genomic “contexts” formed by genes functional interactions, reflected by relationships among their spatial expression patterns across the tissue. Such genomic contexts not only provide insights into the molecular mechanisms underlying biological processes and disease development [9], but also allow the representation of genes as latent manifolds, resembling the learning of word representations from word contexts in linguistic models [1, 10, 11]. These gene representations provide a foundation for quantitatively characterizing context-specific gene functions and interactions from a spatial perspective, facilitating various analytical endeavors where insights into spatial genetic mechanisms are critical.

However, to our knowledge, there lacks gene representation learning approach that leverages ST data to generate spatially-informed gene representations, largely due to the challenges in effectively identifying spatial genomic contexts and encoding spatial gene expression patterns. While recent works, including Gene2Vec [1], scGPT [10], scFoundation [12], scBERT [13], and geneFormer [14], have been developed to learn gene embeddings from atlas-scale microarray or scRNA-seq data, they do not extend to ST, overlooking crucial spatial gene expression information. Moreover, it has been observed that the irreconcilable heterogeneities in their pretraining data collected across a variety of technical and biological conditions may hinder the model’s ability to grasp gene nuances across contexts, leading to suboptimal performance for downstream context-specific tasks [15]. In addition, the high costs of data collection and model pretraining associated with foundational models pose significant challenges for the broader research community in updating these models.

To bridge this gap, we develop a “*context-aware, self-supervised Spatially Informed Gene Embedding Learning* (SIGEL)” framework. SIGEL features in utilizing spatial genomic context inherent in ST data to generate gene manifolds that accurately represent the condition-specific spatial functional and relational semantics of genes. Technically, SIGEL treats gene spatial expressions as images and leverages a masked-image model (MIM), which excels in extracting local-context perceptible and holistic visual features [16], to yield initial gene embeddings. These embeddings are iteratively refined through a self-paced pretext task aimed at discerning genomic contexts by contrasting spatial expression patterns among genes, drawing genes with similar spatial expressions closer in the latent manifold space, while distancing those with divergent patterns. This step enhances the discriminability of gene embeddings, facilitating their incorporation of intricate relational structures among genes. The innovative integration of MIM with contrastive learning equips SIGEL with a superior capability to comprehend and translate spatial gene information, including both expression patterns and inter-gene relationships, into **S**IGEL-generated **G**ene **R**epresentations (**SGR**).

Our study is organized as follows: Firstly, to establish the validity of our method, we demonstrate SIGEL’s effectiveness in identifying gene groups with spatial co-expression patterns, justifying their role as genomic contexts by showing their biological relevance. In addition, the functional and relational semantics of SGRs undergo rigorous validations. Particularly, we demonstrate SGRs’ robustness to technical variations and their utility in gene alignment and comparison across samples. Secondly, we demonstrate the applicability of SIGEL and SGRs in key downstream tasks: i) the batch effects-robust identification of disease-associated genes and gene crosstalk across multiple samples; ii) the de novo imputation of missing genes in FISH-based ST data to expand the transcriptomic coverage of FISH-based ST data, addressing a longstanding challenge that has restricted the broader application of FISH-based ST data; iii) pinpointing genes with designated spatial expression patterns in tissues; iv) detecting spatially variable genes (SVGs); v) improving spatial clustering. Extensive real data analyses demonstrate that SGRs either provide optimal solutions to challenges that have been not inadequately addressed, e.g., the first three tasks, or outperform established benchmarks as seen in task iv and v. These tasks exemplify how SGRs can be effectively employed to address various downstream task-specific objectives, promising SIGEL’s potential in developing a genomic “language”-based methodological ecosystem.

## Results

### Overview of SIGEL

**Fig. 1:**
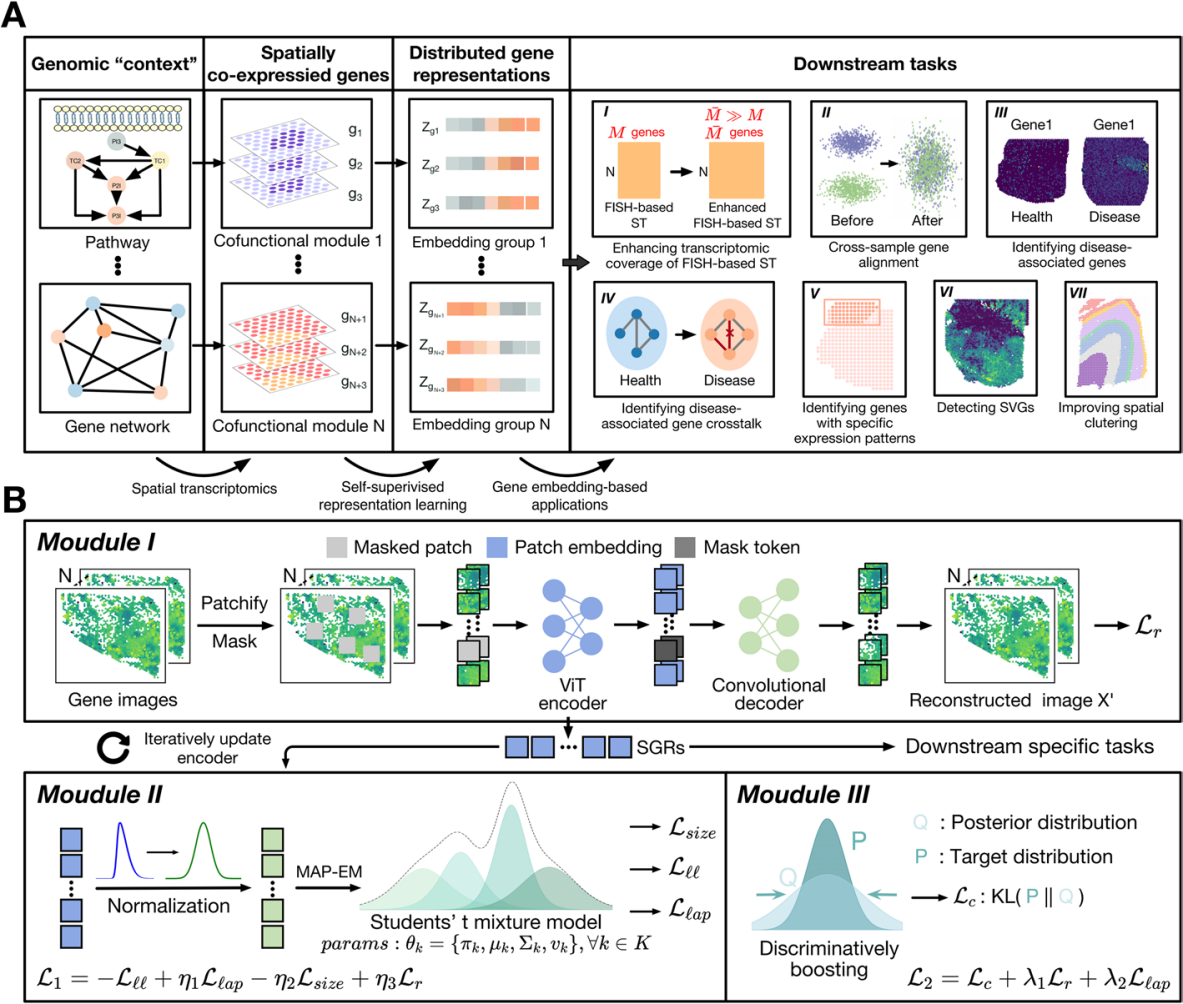
Overview of distributed gene representation learning with SIGEL. **A** Learning distributed gene representations. Spatial transcriptomics (ST) data provide spatial genomic contexts, uncovering genes with cofunctional roles in pathways and networks through their similar expression patterns across tissue space. SIGEL leverages these spatial contexts to derive distributed gene representations that encapsulate spatial functional and relational semantics, thereby enabling a wide range of downstream analyses. **B** Workflow of SIGEL. SIGEL comprises three modules: In *Module I* SIGEL employs an adapted masked autoencoder (MAE) to learn the representations of gene images, which enhances gene embeddings’ local-context perceptibility. *Module II* involves modeling gene embeddings using a Student’s t mixture model, with parameters estimated via a MAP-EM algorithm. This module aims to maximize the likelihood of the entire dataset. In *Module III*, gene embeddings are refined through a self-paced pretext task that aims to identify genomic contexts via iterative pseudo-contrastive learning. Together, modules II and III constitute a single training epoch, in which gene embeddings’ discriminability is enhanced. The training process continues until either the change in gene assignments falls below a threshold or a predetermined number of epochs is reached. Upon training completion, SIGEL-generated gene embeddings (SGRs) can be utilized in downstream analytical tasks, such as i) enhancing transcriptomic coverage of FISH-based ST, ii) cross-sample gene alignment, iii) identifying disease-associated genes, iv) identifying disease-associated gene crosstalk, v) pinpointing genes with designated spatial expression patterns, vi) detecting SVGs, vii) improving spatial clustering

The fundamental idea (Fig. 1A) of SIGEL is that genes co-functional in gene networks and pathways typically exhibit similar spatial expression patterns in tissues, forming a “genomic context” resembling the word context in natural languages. Through a self-supervised learning of the “proximity” of genes in spatial transcriptional activity, as implied by the ST data, we can concurrently identify spatial genomic contexts and acquire semantically meaningful gene embeddings in a data-driven manner. These embeddings, representing spatial gene functions and relationships, can facilitate downstream task-specific objectives. Specifically, as illustrated in Methods and Fig. 1B, SIGEL mainly consists of three modules. In *module I*, we leverage an adapted masked autoencoder (MAE) [16] to transform gene images into gene embeddings that follow a mixture of multivariate Student’s t distributions (see “Representation learning of spatial gene expression maps” in Methods). With this MIM, SIGEL gains the local-context perceptibility by learning to regenerate masked image patches from the surrounding contexts. In *module II*, the gene embeddings are modeled as a Students’ t mixture model (SMM) in the latent feature space, with each mixture component serving as a genomic context comprising spatially co-expressed genes. After the estimation of SMM parameters via a Maximum a posterior (MAP)-EM algorithm, soft assignments of genes to the mixture components are computed (see “SMM-based modeling” in Methods). *Module III* implements a semi-contrastive learning process, through which SIGEL gains the ability to discriminate between spatial gene expressions and grasps the intricate relational semantic structures among genes. This process involves a self-paced, iterative joint optimization of MAE weights and SMM parameters using two loss functions $\mathcal {L}_{1}$ and $\mathcal {L}_{2}$ (see “Self-paced semi-contrastive optimization of gene embeddings” in Methods). Each training epoch begins with $\mathcal {L}_{1}$, updating the MAE weights to maximize the log likelihood of the entire dataset while controlling for macro-factors (e.g., cluster size imbalance) via regularization terms. Following this, $\mathcal {L}_{2}$, designed for discriminatively boosted clustering, further refine gene embeddings and SMM parameters, drawing closer similar genes and distancing dissimilar ones over successive batches. Overall, the training process alternates between the module II and III until either a predetermined number of training epochs is reached, or the change in gene assignments between successive epochs falls beneath a prespecified threshold.

### SIGEL identifies spatially co-expressed gene clusters as biologically relevant genomic context

The effectiveness of SIGEL in generating semantically meaningful gene representations hinges on its ability to identify biologically relevant genomic contexts, manifested as clusters of spatially co-expressed genes. To systematically evaluate this ability, we utilize twelve human dorsolateral prefrontal cortex (DLPFC) 10x Visium datasets (10x-hDLPFC)[17] and a mouse hippocampus Slide-seqV2 dataset (ssq-mHippo) [18], as listed in Additional file 2: Table S1. We benchmark SIGEL against four state-of-the-art competing methods: CNN-PReg [19], Giotto [20], Spark [21] and STUtility [22] (Additional file 2: Table S2). Our initial assessment involves visualizing spatial expression maps of four randomly selected genes from each of two SIGEL-identified clusters, chosen to represent high and medium quality clusters, respectively (see “*Identifying groups of spatially co-expressed genes*” in Additional file 3: Note 1.1). Additional file 1: Fig. S1 shows that genes within both clusters exhibit congruent expression patterns. Next, the overall co-expression and spatial coherence of SIGEL-generated clusters are quantitatively measured using two Davies-Bouldin (DB) indices (see “*Evaluation metrics*” in Additional file 3: Note 1.2). Additional file 1: Fig. S2A shows that SIGEL consistently outperforms the competing methods in both DB indices, demonstrating its effectiveness in identifying spatially co-expressed gene clusters.

To verify the legitimacy of using SIGEL-identified gene clusters as spatial genomic contexts, we delve into their biological significances through gene pathway enrichment analysis and gene cofunction analysis using the 10x-hBC dataset derived from human breast cancer tissue [19]. Our analysis targets three specific gene clusters (C1, C2, and C3), each comprising over 20 genes and demonstrating the lowest group closeness centrality (Additional file 3: Note 2). This centrality metric suggests their expression patterns are most likely to diverge from the majority, probably due to pathological functions. As depicted in Additional file 1: Fig. S2B, the aggregated expression patterns [23] (Additional file 3: Note 3) of C1 through C3 are associated with the spatial distributions of invasive ductal carcinoma (IDC), ductal carcinoma in situ (DCIS) and benign stroma cells, respectively. In contrast, the aggregated expression of C4, a control cluster comprising 30 randomly selected genes, is dispersed over the spatial map. Additionally, Additional file 1: Fig. S2C-D and Additional file 2: Table S3 showcase that these clusters are statistically significantly enriched with pathologically/biologically relevant and densely inter-connected gene ontology biological processes (GOBP). The functional coherence of member genes within these clusters is also notable, given the involvement of a gene in a GOBP can be reliably predicated based on other member genes’ involvement in the same GOBP (see Additional file 1: Fig. S2C). A more detailed explanation of the methodology and results of this analysis is available in the “*Experimental settings*” and Additional file 3: Note 4. These findings altogether affirm that SIGEL-identified gene clusters are cofunctional and biologically/pathologically relevant to the context under investigation, thus endorsing their roles as spatial genomic contexts.

### SGRs effectively capture fundamental gene semantics

We begin this section by validating that SGRs possess superior capabilities in capturing functional and relational semantics through four analyses (details can be found in “Evaluating SIGEL-generated gene embeddings” in Additional file 3: Note 1.3). In these analyses, SGRs are compared against gene embeddings generated by seven state-of-the-art (SOTA) methods. To ensure a fair comparison, all embeddings are derived from the 10x-hDLFPC-151676 dataset. Based on their treatment of genomic context, SIGEL and the benchmark methods are categorized into three groups: (i) models that incorporate spatial genomic context, including SIGEL and stFormer [24]; (ii) models that consider nonspatial genomic context, such as scNET [25], Gene2Vec [1], Geneformer v2 [14], scGPT [10], and scBERT [13]; and (iii) models without considering genomic context, such as DCA [26].

**Fig. 2:**
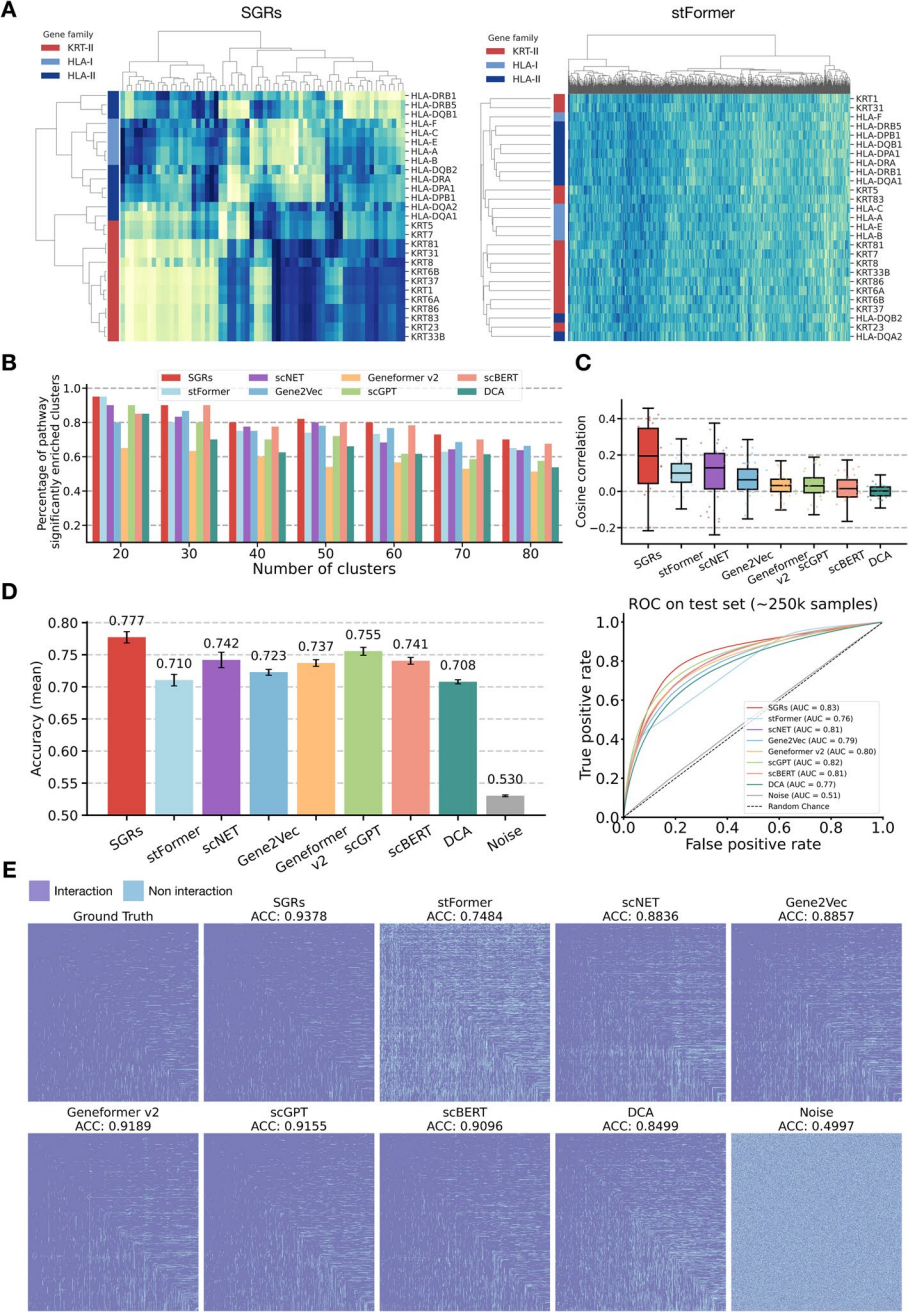
Validating the relational semantics of SGRs. **A** Hierarchical clustering of HLA-I, HLA-II, and KRT-II gene families based on SGRs (left panel) and stFormer embeddings (right panel). The y-axis displays gene family members ordered by hierarchical clustering of SGRs. The x-axis represents the dimensions of the respective embeddings. Gene families are indicated by colors on the y-axis. Blue color intensity positively correlates with the embedding values. **B** Percentage of pathway-enriched gene clusters as a function of the number of clusters. **C** Box plots of GO semantic similarity values for gene embeddings obtained from different methods. **D** Predictive performance metrics for gene-gene interaction predictions using different types of gene embeddings. This panel showcases the mean accuracies (left) and ROC AUC scores (right) from Gene-Gene Interaction Predictor Neural Network (GGIPNN)-based predictions using gene embeddings from SIGEL, stFormer, scNET, Gene2Vec, Geneformer, scGPT, DCA, and randomly generated embeddings. Refer to Additional file 3: Note 8.3 for details on dataset creation. **E** Gene-gene interaction heatmaps comparing ground truth with prediction results. Heatmaps are presented for the ground truth (top-left) and predictions from representative gene embeddings. For better visualization, heatmaps include only the top 1,000 genes with the most interactions according to ground truth. In these maps, a filled cell indicates the existence of an interaction between the corresponding pair of genes, while a blank cell indicates the absence of an interaction. The prediction accuracy is indicated above each prediction heatmap

In the first analysis, we performed hierarchical clustering to the eight gene embeddings and the original expression profiles derived from members of the KRT-II, HLA-I, and HLA-II gene families in human DLPFC tissue (Fig. 2A, Additional file 1: Fig. S3, Additional file 2: Table S4). The results showed that SGRs from the same family clustered well together, and those from functionally related gene families (e.g., HLA-I and HLA-II) were positioned in closer proximity within the hierarchy than those from less related families (e.g., KRT-II and HLA-II). In contrast, when using gene embeddings obtained by other methods, the distinguishability of gene families was significantly reduced as they frequently intermixed along the hierarchical tree. In the second analysis, we used the Leiden [27] clustering algorithm to cluster gene embeddings at various resolution levels by setting the target number of clusters from 20 to 80. For each resolution, we performed pathway enrichment analysis using the *Reactome* database and calculated the proportion of clusters significantly enriched for at least one biological pathway. As shown in Fig. 2B, SGR-derived clusters consistently yielded higher proportions of enriched clusters than those produced by benchmark embeddings, indicating that SGRs more effectively capture biologically meaningful relationships. The third analysis assessed whether SGRs encode Gene Ontology (GO)-based functional similarity. We computed GO Resnik similarities for each gene pair [25, 28], which measures their semantic similarity based on the location of their most informative common ancestors in the GO hierarchy. Meanwhile, cosine similarities of each gene pair are computed using SGRs and the seven benchmark embeddings. A higher correlation between these two similarity measures indicates stronger alignment with GO-defined functional semantics. As shown in Fig. 2C, SGRs achieved the highest average correlation ($\sim$0.18), while DCA, which overlooks genomic context, performed the worst ($\sim$0.01), highlighting the importance of spatial genomic context in learning biologically meaningful embeddings. The fourth analysis predicts gene-gene interactions using SGRs, the seven benchmark embeddings, and a set of randomly generated embeddings. As detailed in Additional file 3: Note 5, SGRs outperformed all benchmarks in terms of prediction accuracy and AUC (Fig. 2D). Moreover, the interaction heatmap generated from SGRs more closely resembled the ground truth than those generated by baseline methods (Fig. 2E). Collectively, these analyses demonstrate SIGEL-derived SGRs outperform benchmark methods in capturing both functional and relational gene semantics.

### SGRs facilitate cross-sample gene alignment

**Fig. 3:**
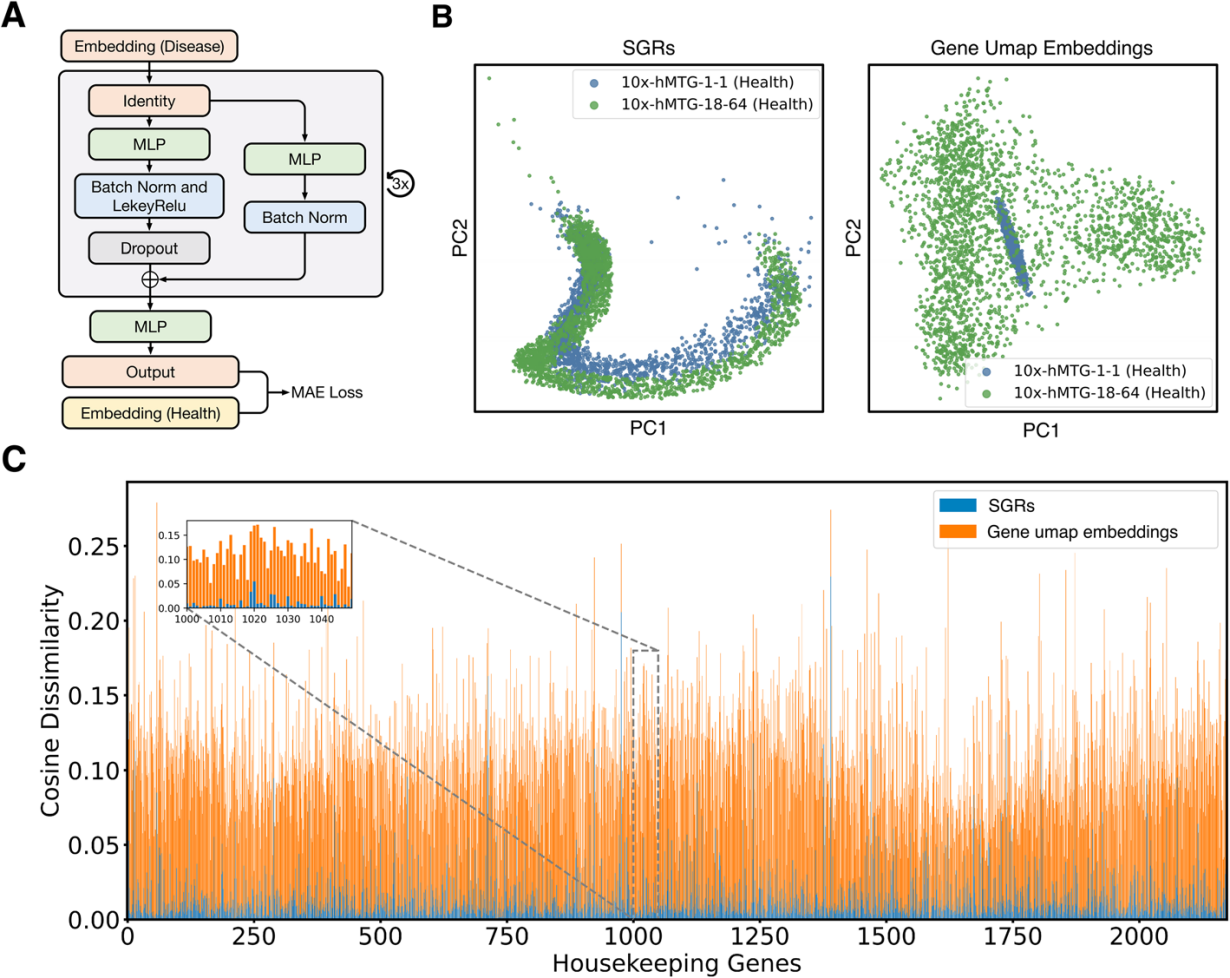
Cross-sample gene alignment with SGRs. SGRs of 2177 housekeeping genes are generated from two healthy human MTG 10x Visium dataset (10x-hMTG-1-1 and 10x-hMTG-18-64), respectively. UMAP embeddings of the same dimension as SGRs for these housekeeping genes are also generated from both datasets. Embedding pairs of identical genes but from different datasets are then subjected to alignment. **A** The network architecture of SGR alignment network (SAN). **B** PCA plots of SGRs (left) and gene UMAP embeddings (right) after SAN-mediated alignment. **C** Scaled cosine dissimilarities between pairs of aligned SGRs (in blue) versus those between aligned UMAP embeddings (in orange)

Given that gene-gene relationships remain relatively stable across datasets under identical conditions and are less prone to technical artifacts like batch effects [29], we posit that SGRs, generated based on gene relational semantics as previously validated, are also resilient to technical artifacts, thus capable of facilitating cross-sample gene alignment. To verify this point, SGRs from two healthy human middle temporal gyrus (MTG) 10x Visium datasets (10x-hMTG-1-1 and 10x-hMTG-18-64) are aligned using our SGR alignment network (SAN), which is a three-layer feedforward neural network (FFN) with nonlinear activation functions (Fig. 3A). SAN learns a mapping function, ***F***, that minimizes the mean absolute errors between SGR pairs of 2177 housekeeping genes from two different datasets (see “data availability” for where housekeeping genes are acquired). The consistent biological roles of these housekeeping genes suggest that their SGRs should align accurately if they are unaffected by cross-sample technical variations. For comparison, gene expressions from both datasets are also transformed into uniform manifold approximation and projection (UMAP) embeddings with the same dimensionality as SGRs. SAN is then trained to align these UMAP embeddings, which could potentially be confounded by technical variations. The cosine dissimilarity of aligned embeddings pairs serves as an indicator of alignment discrepancies. Figure 3C reveals that discrepancies between SGR pairs are significantly lower than those between pairs of UMAP embeddings, highlighting SGRs’ robustness against cross-sample technical noise. Moreover, by visualizing aligned SGRs and UMAP embeddings on a principal component analysis (PCA) plot (Fig. 3B), we find SGRs from different datasets are more evenly mixed compared to the UMAP embeddings. These observations provide strong evidence to that SGRs prioritize capturing genuine gene semantics and are resistant to technical artifacts across samples, thereby facilitating more accurate cross-sample gene alignment.

### Identifying disease-associated genes and gene crosstalk with SGRs

Identifying genes with altered spatial expression and interactions is pivotal for illuminating pathogenic mechanisms underlying disease progression, e.g., the elevated APOE expression within hippocampus in Alzheimer’s Disease (AD) [30] and the intensified interplay between Notch and Wnt pathways in many cancers [31]. We define strategies for identifying disease-related genes as either reference-based or reference-free. The former compares spatial gene expressions in diseased versus healthy tissues to pinpoint differences, while the latter identifies genes exhibiting specific expression patterns within putative pathogenic regions independently of healthy tissue expression benchmarks. Herein, we detail the use of SIGEL and SGRs in both strategies.

*Reference-based*. In ST, directly comparing gene expression patterns between conditions is difficult due to variations in tissue slice preparations, technical artifacts, and spatial heterogeneity across slices. Nevertheless, SGRs, which are context-aware embeddings capturing fundamental gene semantics and whose value discrepancies across samples/conditions are reconcilable by SAN-mediated gene alignment as shown in Fig. 3A, allow for the detection of disease-associated alterations in spatial gene expressions and relationships.

**Fig. 4:**
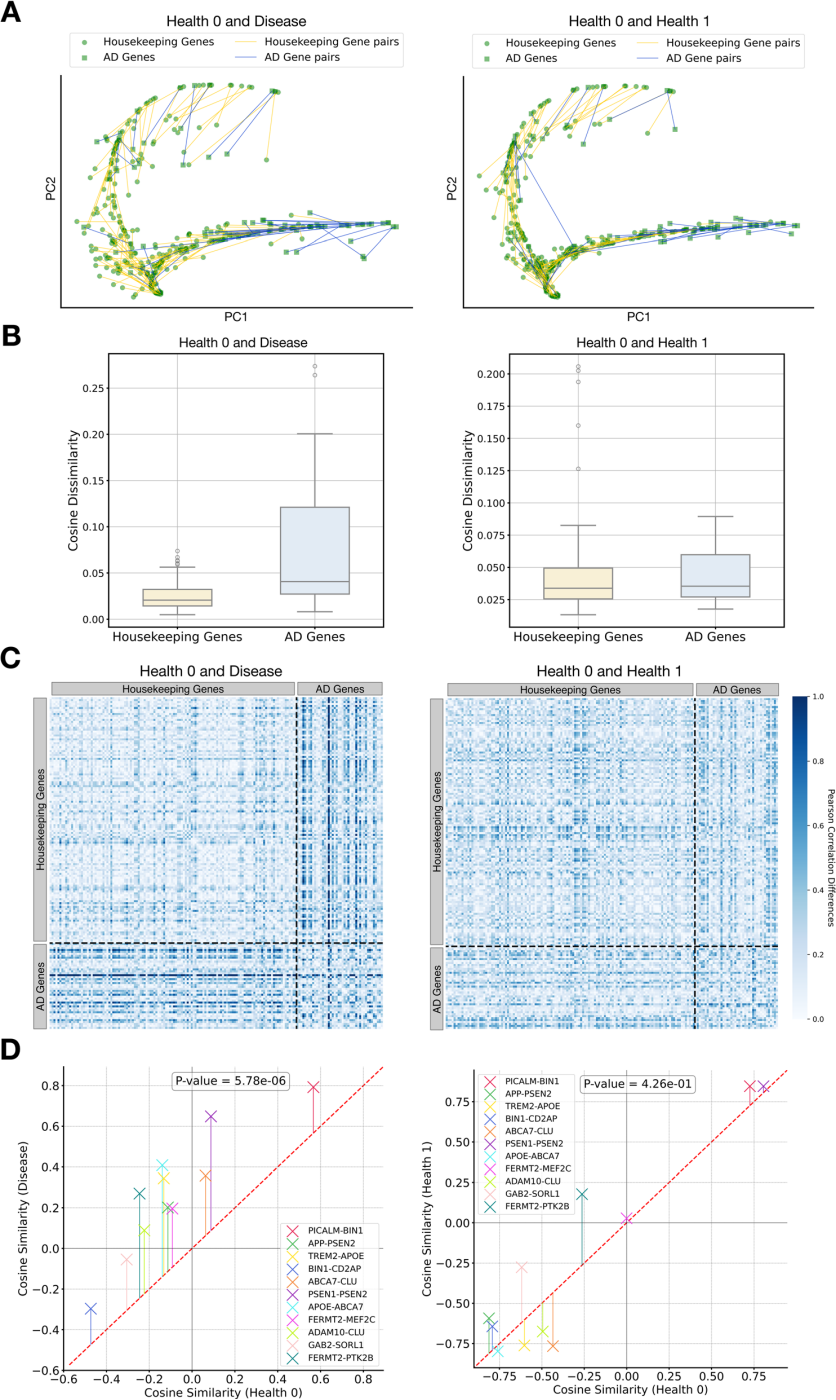
Identifying AD-associated genes and gene crosstalk with SGRs. **A** and **B** are for identifying AD-associated genes, while **C** and **D** for AD-associated gene crosstalk. SGRs of 42 AD-associated genes and 126 non-anchor housekeeping genes are obtained from two healthy MTG 10x Visium datasets (health_0: 10x-hMTG-1-1 and health_1: 10x-hMTG-18-64) alongside an AD MTG 10x Visium dataset (10x-hMTG-2-3). SGR pairs of identical genes but from different datasets are aligned using SAN. The AD and the healthy (health_0) datasets form the study group, while the two healthy datasets form the control group. **A** PCA plots of SGRs in the study (left) and control (right) groups. Round dots and triangles represent housekeeping and AD-associated genes, respectively. The PCA distances between SGR pairs are visually represented by yellow and blue lines for housekeeping and AD-associated genes, respectively. Compared to the control group, blue lines are markedly longer than yellow lines in the study group. **B** Box-plots of scaled cosine dissimilarities between SGR pairs in the study (left) and control (right) groups. Yellow and blue boxes represent housekeeping genes and AD-associated genes, respectively. **C** Gene-gene interactions within each dataset are quantified using a Pearson correlation matrix calculated from SGRs. Alterations in gene-gene interactions between two datasets are measured as a correlation shift matrix representing the absolute differences between the two correlation matrices, which is visualized as a heatmap wherein darker colors indicate larger shifts. Compared to the control group (right), correlation shifts in the study group (left) for gene pairs involving at least one AD-associated gene are significantly larger than those for gene pairs of housekeeping genes only. **D** Scatterplots of correlations between gene pairs involved in the same AD-associated pathways, with each cross representing a gene pair. For the study group (left), y- and x- axes denote gene correlations in the AD and healthy (health_0) datasets, respectively. In the control group (right), these axes represent gene correlations in the health_1 and health_0 datasets, respectively. A t-test is used to assess the statistical significance of differences in gene correlations between the datasets, with P-values indicated on top of each panel

To verify this, we generate SGRs from two healthy brain tissue datasets (10x-hMTG-1-1 and 10x-hMTG-18-64) and one AD brain tissue dataset (10x-hMTG-2-3). SAN is used to align SGR pairs of identical genes from two different datasets. The 2177 housekeeping genes are randomly split into an “anchor” set of 2051 genes for SAN training and a “non-anchor” set of 126 genes reserved for testing. The trained SAN is applied to align SGR pairs from both the non-anchor housekeeping gene set and a set of 42 AD-associated genes reported by previous studies (Additional file 2: Table S5). When aligning SGRs between an AD and a healthy dataset (i.e., 10x-hMTG-1-1), we find that AD-associated genes demonstrate significantly greater PCA distances (Fig. 4A, left) and scaled cosine dissimilarities (Fig. 4B, left) compared to the non-anchor housekeeping genes. In contrast, alignment of SGRs between the two healthy datasets does not show such disparities, neither in PCA distances (Fig. 4A, right) nor in scaled cosine dissimilarity (Fig. 4B, right), aligning with expectations that biological semantics of AD-related genes should remain unchanged across healthy conditions. These findings imply that differences in SGRs between healthy and diseased states could signal changes in gene functions associated with the disease. Therefore, disease-associated genes can be prioritized based on the degree of their SGR dissimilarities.

SGRs can also provide valuable clues to altered gene crosstalk in disease. We denote the Pearson correlations between SGRs of gene pairs from a healthy brain tissue dataset (10x-hMTG-2-5) as $\rho _{\text {health}}$, and from an Alzheimer’s Disease (AD) dataset (10x-hMTG-2-3) as $\rho _{\text {AD}}$. The left panel in Fig. 4C reveals that the absolute difference between these correlations, $\delta _{\rho } = \left| \rho _{\text {health}} - \rho _{\text {AD}} \right|$, signals potential alterations in gene crosstalk, as $\delta _{\rho }$ values are notably higher when at least one gene in the pair is AD-associated compared to pairs of housekeeping genes. This observation is bolstered by a statistically significant increase (P-value=5.78e-06) in correlations between gene pairs that participate in the same AD-related pathways (Fig. 4D, left) in the AD dataset. In contrast, no such shifts in correlation are detected between the two healthy datasets (right panels in Fig. 4D), in line with the expectation that gene relationships in health conditions should remain largely stable. Therefore, substantial changes in SGR correlations are indicative of disease-related alterations in gene interactions.

**Fig. 5:**
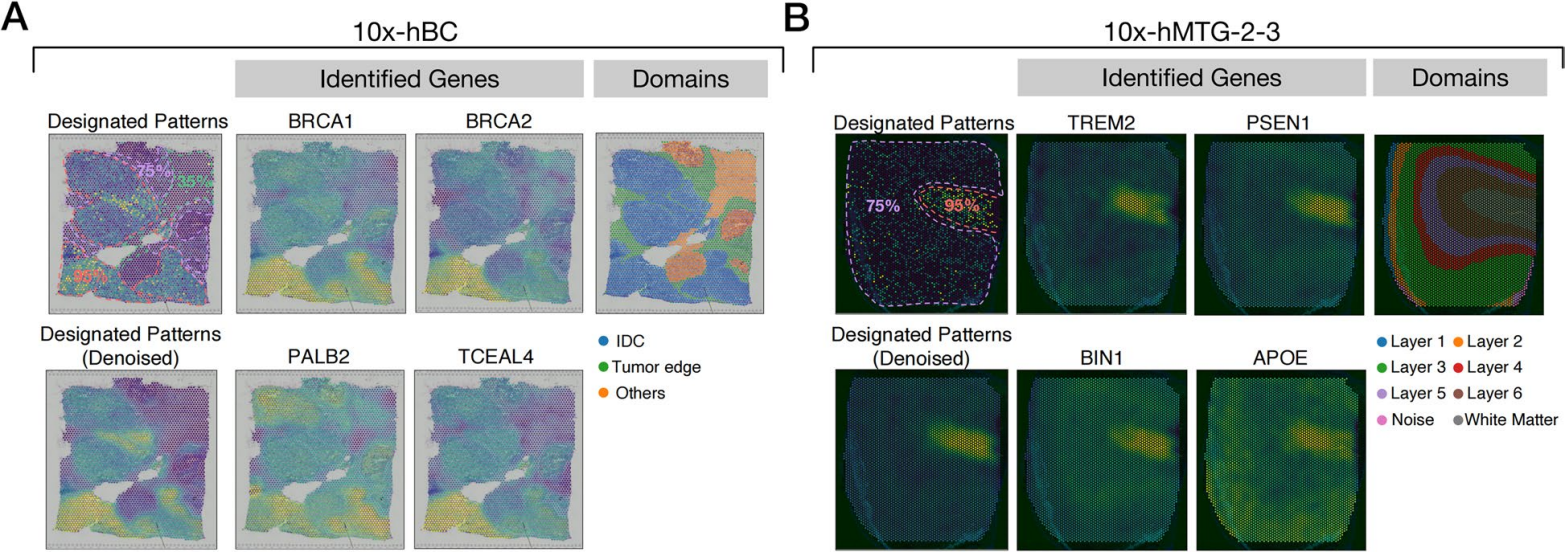
Identifying disease-associated genes with designated spatial expression patterns. For both (**A**, **B**), the designated gene spatial expression patterns are displayed in the leftmost panel in the first row. The expression level percentiles are indicated within different regions demarcated by dotted lines of varying colors. The rightmost panel in the first row shows expert-curated domain labels. The leftmost panel in the second row represents denoised SIGEL-SPS-simulated genes that mirror the designated patterns. The rest panels correspond to denoised spatial maps of identified real genes whose expression patterns resemble the designated ones. **A** The designated expression patterns have a high expression level (95% percentile) in tumor cores (i.e., IDC), a medium expression (75%) in tumor edges, and a low expression (35%) elsewhere in the human breast cancer 10x Visium dataset (10x-hBC). **B** The designated expression patterns have a high expression level (95% percentile) in white matter (WM), a medium expression (75%) in other cortex layers in the human MTG 10x Visium dataset (10x-hMTG-2-3)

*Reference-free*. Our reference-free approach involves employing an innovative method, SIGEL-SPS, to create a pseudo-gene with designated spatial expression patterns, subsequently converted into an SGR. Based on the scaled cosine similarities between the SGRs of real genes and the pseudo-gene, we can pinpoint genes whose spatial expression patterns correspond to the predefined one. Our method is tested on two datasets: a human breast cancer dataset (10x-hBC) and an AD MTG dataset (10x-hMTG-2-3). In the SIGEL-SPS simulation, “high”-level of gene expression is defined as the 95th percentile of average expressions across all genes, “medium”-level the 75th percentile, and “low”-level the 35th percentile. For the 10x-hBC dataset, we aim to discover genes with high expression within tumor cores (IDC), medium expression within tumor edges, and low expression elsewhere. Using SIGEL-SPS, a gene mirroring these expression patterns is simulated. As shown in Fig. 5A, we successfully identify cancer-associated genes such as *BRCA1*, *BRCA2*, *PALB2*, and *TCEAL4*, all exhibiting the sought-after expression patterns. Similarly, for the 10x-hMTG-2-3 dataset, we pinpoint AD-associated genes like *TREM2*, *PSEN1*, *BIN1*, and *APOE*, characterized by high expression within the white matter (WM) layer and medium expression within other cortex layers (Fig. 5B). Particularly, the upregulation of *BIN1* within the WM of AD brain has been previously reported [32]. These outcomes collectively demonstrate the efficacy of utilizing SGRs and SIGEL-SPS for discerning genes with disease-specific spatial expression profiles. Moreover, our methodology extends beyond disease-associated gene identification to encompass any genes with designated spatial expression patterns.

### SGR-based enhancement of the transcriptomic coverage in FISH-based ST

A significant challenge in ST is achieving both full transcriptomic coverage and high-resolution. Existing methods like Tangram [33], SpaGE [34], SpaOTsc [35], stAI [36], and SpatialScope [37] augment transcriptomic coverage in high-resolution, FISH-based ST data, but they essentially generate “pseudo-ST” data since they focus on mapping single cells in scRNA-seq onto spatial locations in ST to compensate for genes not profiled in ST with scRNA-seq data, rather than the de novo generation of ST data with inherent spatial semantics. These methods are limited by their underutilization of spatial information in the mapping process, as seen in Tangram SpaGE, and stAI, and the introduction of systematic biases from discrepancies between scRNA-seq and ST data such as inconsistencies in data scales.

**Fig. 6:**
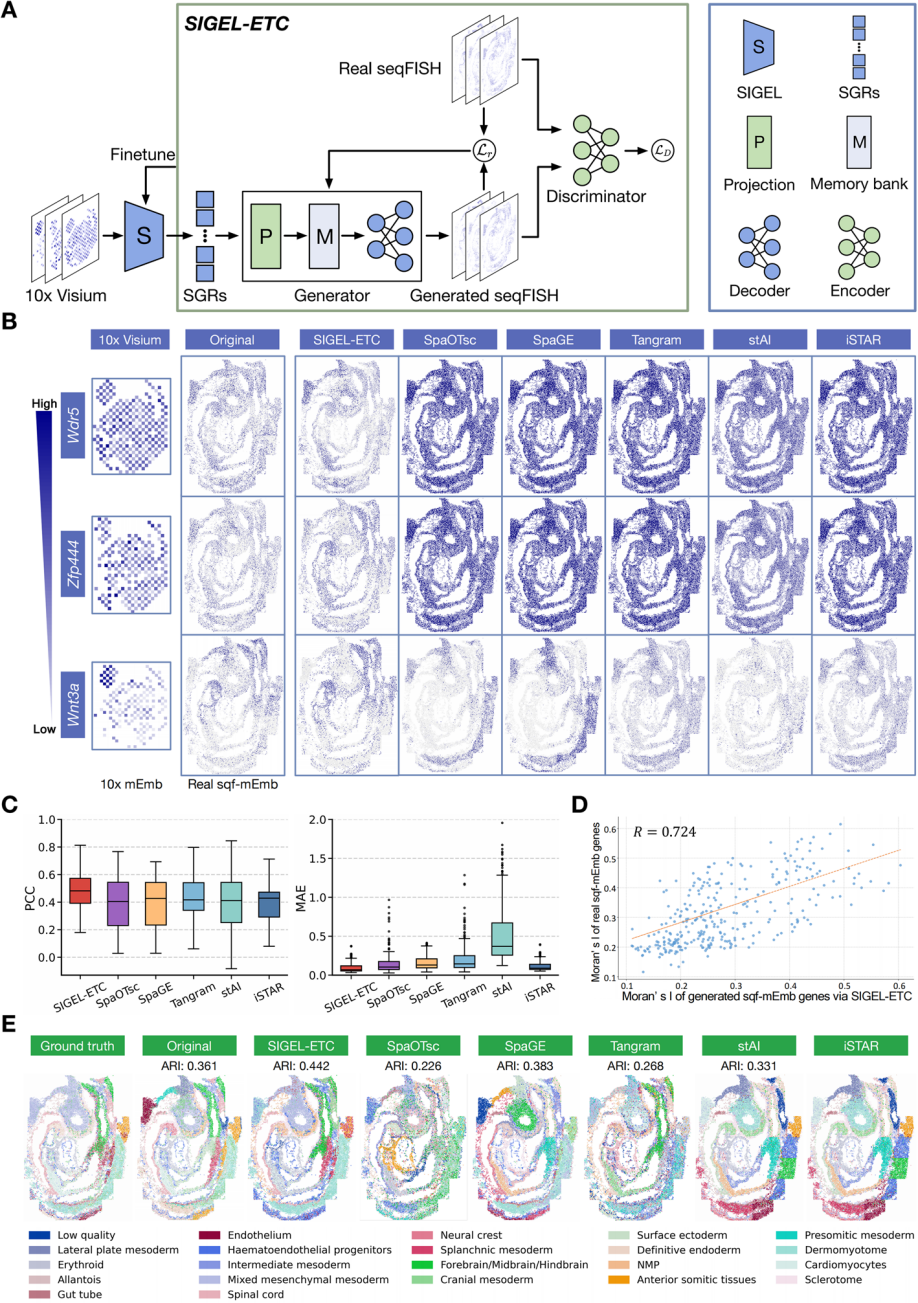
SGR-based enhancement of transcriptomic coverage of FISH-based ST with SIGEL-ETC. **A** Workflow of SIGEL-ETC. Initially, genes from a ST dataset with full transcriptomic coverage are encoded into SGRs. In the training phase, SGRs of the genes present in the target FISH-based ST dataset are fed into SIGEL-ETC’s generator to regenerate their original spatial gene expressions. The generator consists of three components: a multilayer perceptron (MLP)-based encoder, an MLP-based decoder, and a memory bank that ensures the training stability. Following this, a discriminator is trained to distinguish between the original and regenerated genes. Meanwhile, the loss gradients from the generator are backpropagated to update the parameters of the MAE’s encoder within SIGEL, so that SGRs are more adapted to the specific semantics inherent in the FISH-based ST dataset. Once trained, SIGEL-ETC can generate genes that are initially absent in the dataset, using three finetuned SEGs. **B** Three genes (Wdr5, Zfp444, and Wnt3a), which are covered by both the SeqFish (sqf-mEmb) and the 10x-mEmb datasets and differ in their expression levels, are used to evaluate SIGEL-ETC. These genes’ SGRs are initially obtained from the 10x-mEmb dataset and used to regenerate their expression profiles in the sqf-mEmb dataset. From the left to the right, we show the three genes’ original spatial expression profiles in the 10x-mEmb dataset, in the sqf-mEmb dataset, alongside their regenerated expression profiles by SIGEL-ETC and the five benchmark methods. **C** Displayed in the box plot are the Pearson correlation coefficients and mean absolute errors between the original and spatial expression profiles of the 330 genes regenerated by SIGEL-ETC and the benchmark methods. **D** The scatterplot shows spatial variabilities of the 330 genes and their respective regenerated counterparts, with spatial variability quantified using Moran’s I index. The red line in the scatterplot represents a fitted regression line with $R = 0.724$. **E** The transcriptomic coverage of sqf-mEmb is doubled by using SIGEL-ETC and the benchmark methods to generate an additional 330 genes absent in the original dataset. The leftmost spatial map shows the ground truth tissue domain annotations, the second map shows the spatial clustering results of SpaGCN using the unexpanded gene set, while subsequent maps show the spatial clustering results using gene set augmented by SIGEL-ETC and the benchmark methods, respectively. The clustering accuracy (i.e., ARI) is shown below each method name

To address this challenge, we introduce SIGEL-enhanced-transcriptomics-coverage (SIGEL-ETC), an innovative SGR-based Generative Adversarial Network (GAN) model as detailed in the “SIGEL-ETC” in Methods and Fig. 6A. SIGEL-ETC is predicated on the notion that gene relational semantics should remain largely consistent across different ST data types for the same tissue type. Consequently, the spatial expression profiles of uncovered genes can be extrapolated from those of covered genes, drawing on their semantic relationships inherent in SGRs derived from a full transcriptomic coverage ST dataset (e.g., 10x Visium). We evaluate the effectiveness of SIGEL-ETC by reproducing the 330 covered genes from a mouse embryo SeqFISH dataset (sqf-mEmb), guided by the SGRs from a mouse embryo 10x Visium dataset (10x-mEmb). Tangram, SpaGE, SpaOTsc, stAI, and SpatialScope are included as benchmarks (Additional file 2: Table S2) to reproduce the same gene set from scRNA-seq data. To ease a direct comparison between the original and reproduced genes, we visualize the spatial maps of three genes differing in their expression levels, including *Wdr5*, *Zfp444*, and *Wnt3a*. Figure 6B illustrates that SIGEL-ETC surpasses benchmark methods in accurately reproducing genes, achieving high fidelity in both spatial expression patterns and data scales. Additionally, Fig. 6C quantitatively demonstrates SIGEL-ETC’s superiority in generating genes that closely align with their actual values, in terms of both correlation coefficients and mean absolute errors. This concordance is further supported by the highly correlated spatial variability (R=0.724) between authentic and SIGEL-ETC-generated gene expressions, as depicted in Fig. 6D. Finally, we select an additional 330 genes imputed by SIGEL-ETC, deemed most real-like by the GAN discriminator, to double the transcriptomic coverage of the sqf-mEmb dataset. This augmented gene set undergoes spatial clustering to evaluate the imputed genes’ quality and their analytical utility. Figure 6E shows that spatial clustering with SIGEL-ETC-imputed genes achieves a significantly higher accuracy compared to either the original dataset or genes imputed by the benchmark methods. Collectively, these results highlight the efficacy of SGRs in enhancing the transcriptomic coverage of FISH-based ST via a generative approach.

An alternative strategy for gene imputation involves predicting gene expression directly from paired histological images, when such images are available. To compare SIGEL-ETC with this histology-guided approach, we adopted iStar [38] and conducted a benchmark using a human breast cancer 10x Visium dataset (10x-hBC-H1) as reference and a human breast cancer Xenium dataset (Xenium-hBC) as target, which consists of both high-resolution FISH-based spatial transcriptomics and the associated histological images. Specifically, iStar was first trained on the reference dataset to predict gene expression from histology images, and then applied to Xenium tissue images to infer high-resolution gene expressions. For SIGEL-ETC, the same training and prediction procedure was conducted as in the sqf-mEmb experiment. We evaluated the performance of iStar and SIGEL-ETC on predicting the expressions of 150 genes common to both the reference and target datasets. Additional file 1: Fig. S4 shows that both iStar and SIGEL-ETC can effectively impute gene expression in the Xenium-hBC dataset, with SIGEL-ETC performing better than iStar (Additional file 1: Fig. S4A), as indicated by its higher Pearson correlation coefficients and lower mean absolute errors across test genes (Additional file 1: Fig. S4B). The superiority likely stems from SIGEL-ETC’s ability to capture spatial gene–gene relationships, enabling it to infer missing genes based on those detected in the FISH dataset. In contrast, iStar imputes the missing genes solely based on histological cues.

### SGR-based SVG detection

In this section, we introduce a novel computational method, SIGEL-SVG, that leverages SGRs to detect SVGs from ST datasets, as detailed in the “SIGEL-SVG” section in Methods. Essentially, SIGEL-SVG calculate a spatial variability score for each gene based on the similarity of its SGR with those of simulated spatially homogeneous genes. SVGs then are ranked and selected according to these scores. For this assessment, we select the top 3000 SVGs from both the 10x-hDLPFC-151507 and 10x-hBC datasets using SIGEL-SVG and three prevalent benchmark methods: SPARK [21], SPARK-X [39], and SpatialDE [40] (Additional file 2: Table S2). Both Moran’s I and Geary’s C indices shows that the SVGs selected from the 10x-hDLPFC-151507 dataset by SIGEL-SVG are more spatially variable than those selected by the benchmark methods (see Additional file 1: Fig. S5A). Moreover, one well-documented drawback of the benchmark methods is their inability to effectively rank SVGs based on their spatial variability scores (*p*- or *q*-values) [41]. In contrast, SIGEL-SVG is more sensitive and effective in distinguishing levels of spatial variability among SVGs. This is exemplified in Additional file 1: Fig. S5B, where the top four SVGs selected by SIGEL-SVG exhibit more noticeable spatial variabilities than those selected by the benchmark methods. A parallel analysis conducted on the 10x-hBC dataset yields similar results (see Additional file 1: Fig. S6). These results altogether demonstrate the potential of SGRs for detecting and ranking SVGs in ST datasets.

### SGR-improved spatial clustering

In this section, we propose SIGEL-Improved-Spatial-Clustering (SIGEL-ISC), a novel SEG-based computational method for enhancing spatial clustering, as detailed in the “SIGEL-ISC” section in Methods. The rationale behind SIGEL-ISC is that spatial clustering can be effectively improved by optimizing the informational efficiency of spatial transcriptomic data, which involves minimizing redundant information and retaining the most discriminative information presented by feature genes [42]. Redundant information among genes can be revealed through their SGR similarity matrix and reduced by only selecting and retaining the most discriminative genes within groups of highly similar ones. The most discriminative genes are those with the highest spatial variability scores, as determined by SIGEL-SVG. Subsequently, any spatial clustering algorithm, which is SpaGCN [41] in our case, can work with this information-efficient set of feature genes to achieve improved performance. We select two state-of-the-art spatial clustering methods, GraphST [43] and SpaGCN, alongside a baseline method, Leiden, as benchmarks for comparison (Additional file 2: Table S2). In a comprehensive evaluation across twelve 10x-hDLPFC datasets, SIGEL-ISC consistently outperforms the benchmark methods, as evidenced by its highest Adjusted Rand Index (ARI) and Normalized Mutual Information (NMI) scores (see Additional file 1: Fig. S7A). SIGEL-ISC’s superiority over the benchmark methods is further illustrated by its more accurately recovered annotated anatomical cortex layers in the spatial maps of the 10x-hDLPFC-151676 and 10x-hDLPFC-151669 datasets (see Additional file 1: Fig. S7B). Finally, SIGEL-ISC achieves optimal performances across six 10x-hDLPFC datasets when approximately 50%−60% redundant information is excluded (see Additional file 1: Fig. S7C).

### Complexity and sensitivity analyses

In this section, we analyze the computational efficiency of SIGEL from both theoretical and empirical perspectives. We also conducted a sensitivity analysis over SIGEL’s key hyperparameters using the 10x-hDLPFC-151676 dataset. All these analyses were conducted using an RTX 3090 GPU (24 GB).

*Complexity analysis.* Theoretically, SIGEL exhibits a computational complexity of approximately *O*(*N*), where *N* is the number of spatial gene maps. Specifically, SIGEL’s self-supervised representation learning component is implemented as a lightweight MAE module with *O*(*N*) complexity [16]. SIGEL’s SMM component also has a complexity comparable to the Gaussian Mixture Model (GMM) with *O*(*N*) complexity [44], due to its closed-form parameter updates. Additionally, SIGEL’s KL-based clustering maintains a complexity of *O*(*N*), as indicated by previous work [45]. To empirically validate this scalability, we measured SIGEL’s runtime across increasing number of genes, from 5,000 to 20,000. As shown in the top panel in Additional file 1: Fig. S8A, the runtime was 4.22, 7.47, 10.58, and 13.72 minutes for 5,000, 10,000, 15,000, and 20,000 genes, respectively, indicating near-linear scalability with respect to the number of gene maps. In terms of resource efficiency, SIGEL required 5.12M parameters and 59.30M FLOPs, compared to 0.45M parameters and 1565.24M FLOPs for scNET (Additional file 1: Fig. S8A, bottom). These results indicate that SIGEL is significantly more computationally efficient than scNET, particularly in terms of FLOPs. Finally, we can trade some flexibility of SIGEL for further improved computational efficiency by fixing the degree of freedom in the Student’s t-distribution to 1. This reduces the distribution to a Cauchy distribution and bypasses the need for gradient-based inference of this parameter.

*Hyperparameter sensitivity analysis.* We conducted a series of sensitivity analyses to assess the impact of four key hyperparameters in SIGEL, including the mask ratio in the MAE module, the dimensionality of the SGR embeddings, the number of SMM clusters, and the reconstruction loss weight $\lambda _1$ in the total loss $\mathcal {L}_2$. Model performance was evaluated based on the quality of gene clustering, using two DB indices as described in “Evaluation metrics” (Additional file 3: Note 1.2). For the mask ratio in the MAE module, we tested values ranging from 20%–90%. Results show that a mask ratio of 80% provides an optimal trade-off between training stability and representation quality (Additional file 1: Fig. S8B, top left). For SGR dimensionality, we varied the embedding size from 16 to 256. While lower-dimensional embeddings are more robust and computationally efficient, they may lack expressiveness; higher dimensions increase capacity but risk overfitting. We found that 64-dimensional embeddings strike the best balance between expressiveness and robustness (Additional file 1: Fig. S8B, top right). The number of SMM clusters in Module II is by default set to approximate the typical number of genes in a KEGG pathway (30 per cluster) [46]. We evaluated values ranging from 50 to 850. As shown in Additional file 1: Fig. S8B (bottom left), performance improves with increasing cluster counts up to 450, stabilizes between 450 and 750 (where the default of 610 lies), and declines beyond that point. Finally, for the reconstruction loss weigh $\lambda _1$, which balances the contribution of local gene contextual semantics (via the reconstruction loss) and global gene semantics (via the KL divergence loss), we tested values from 0.1 to 0.5. Optimal performance was observed at $\lambda _1=0.3$, as shown in the bottom right panel of Additional file 1: Fig. S8B.

## Discussion

In ST, genomic contexts unveiled as groups of spatially cofunctional and co-expressed genes are instrumental for generating semantically rich gene embeddings, paralleling the concept of word vectorization in natural languages processing. Existing foundational models designed to learn gene embeddings from microarray or scRNA-seq data typically rely on massive pretraining, resulting in a weakened sensitivity to context-specific nuances, and fall short of incorporating spatial expression information into the gene vectorization process. In this work, we propose SIGEL, a pioneer context-aware, self-supervised learning model that exploits spatial genomic contexts in ST to derive distributed gene representations imbued with spatial gene functional and relational semantics.

We comprehensively evaluate SIGEL across ST datasets of various tissues, species, and platforms in aspects regarding the model’s legitimacy and its utility in downstream task-specific applications. To establish the methodological soundness, we initially demonstrate SIGEL’s adeptness at identifying spatial genomic contexts as groups of cofunctional and co-expressed genes. Subsequent analyses confirm SIGEL’s ability in generating SGRs that encapsulate essential gene semantics from these genomic contexts, with SGR correlations reflecting gene familial and ontological ties. Notably, SGRs prioritize biological variations over technical noise, enhancing their utility in cross-sample gene alignment, as demonstrated by the accurate alignment of SGRs of functionally stable housekeeping genes. For task-specific applications, we propose a suite of innovative SGR-based methods for identifying disease-associated genes and gene crosstalk, pinpointing genes with designated spatial expression patterns, enhancing the transcriptomic coverage of FISH-based ST, detecting SVGs, and improving spatial clustering. These methods either pioneer solutions to existing problems or markedly surpass established benchmarks.

SIGEL’s remarkable performance are rooted in four aspects: the effective integration of spatial expression patterns into SGRs via an image-focused, self-supervised MIM approach; learning relational semantic structures among genes through a flexible and robust SMM-based clustering; a novel combination of MIM with contrastive learning for iterative joint optimization, enhancing the perceptibility and discriminability of SGRs; and the resilience of SGRs to technical noise, ensuring reliable gene alignment across conditions. Overall, SIGEL not only facilitates the discovery of cofunctional gene modules, like gene networks and pathways, but also generates biologically significant gene embeddings, laying the foundation for a suite of downstream task-specific tools. Thus, SIGEL promises to contribute to the development of a genomic “language”-based methodological ecosystem. Future improvements for SIGEL may include enriching the informativeness of SGRs through a multimodal learning approach, integrating diverse gene relational semantics from additional datasets like gene co-expression patterns across cell types observed in scRNA-seq, and extend the detection of global SVGs to cell type-specific SVGs [47, 48] based on the spatial distribution of the target cell type and the cell type-specific spatial gene maps.

## Conclusions

In this study, we introduced SIGEL, a novel context-aware, self-supervised framework that addresses the critical gap of learning spatially-informed gene representations from ST data. Our comprehensive validations demonstrate that the resulting SGRs are robust, broadly applicable, and effectively capture the functional and relational semantics of genes. The efficacy of SGRs was validated through critical downstream applications, enabling the *de novo* imputation of genes in FISH-based data and facilitating the robust identification of disease-associated genes across samples, while demonstrating superior performance over established benchmarks in tasks such as SVG detection and spatial clustering. These findings underscore SIGEL’s capability to provide deeper mechanistic insights into the spatial organization of gene expression in complex tissues and disease, and to establish a powerful computational foundation for a new ecosystem of genomic “language”-based analyses, promising to accelerate future biological and biomedical discoveries.

## Methods

### Data auality control and preprocessing

We conform to the conventional procedure for preprocessing ST data, as implemented in the SCANPY package [49]. Specifically, we first remove mitochondrial and External RNA Controls Consortium (ERCC) spike-in genes. Then, genes detected in fewer than 10 spots are excluded. To preserve the spatial data integrity, we do not perform quality control on spatial spots. Finally, the gene expression counts are normalized by library size, followed by log-transformation.

### Representation learning of spatial gene expression maps

As the spatial gene maps can be visualized as gray-scale images, we devise an adapted version of MAE to transform visual features of gene images into embeddings in a latent feature space. A given gene image is first segmented into regular non-overlapping patches, from which a subset of patches is randomly selected, masked and discarded. The remaining patches are fed into the MAE encoder to generate visible patch embeddings. Given that a gene image is gray-scale and often sparse, we use a higher masking ratio (80%) and a light-weighted ViT encoder with four transformer blocks and four attention heads rather than the masking ratio (75%) and the ViT-encoder in the original paper. The visible patch embeddings and trainable tokens of masked patches are then input into the MAE decoder to reconstruct the gene image. We replace the transformer architecture of the original MAE decoder with a convolutional autodecoder to enhance the performance in our case. A more important modification is the adding of a nonlinear projection head to the end of the encoder. This projection head consists of a linear layer, a batch normalization (BN) layer and a Scaled Exponential Linear Unit (SELU) activation layer. Owing to the BN layer and the self-normalizing property of the SELU function, the gene embeddings output from the encoder more closely conform to the mixed Student’s t distribution.

### SMM-based modeling

As Stuhlsatz et al [50] have demonstrated the capability of deep image encoder in learning visual representations that follow a multivariate Student’s t-distribution, we utilize an SMM to model the distributions of SGRs in a latent feature space, with individual components of the SMM corresponding to distinct gene clusters. The key strength of this modeling is its robustness to outliers, which are assigned reduced weights during the estimation of model parameters. Specifically, let $Z\in R^{N\times D}$ denote SGRs, where *N* is the total number of genes and *D* is the dimension of the feature space. We model the distribution of Z as an SMM parameterized by $\Theta =$ $\{\Theta _k{:}\pi _k,\mu _k,\Sigma _k,v_k,\forall k\in K\}$. Here, K represents the total number of gene clusters, while $\pi _k,\mu _k,\Sigma _k,\upsilon _k$ represents the weight, mean, covariance matrix and freedom of the *k*-th component, respectively. The density function of $z_i$ is then formulated as follows:

1 $$\begin{aligned} p(z_i|\Theta )=\sum _{k=1}^K\pi _k\Phi (z_i|\mu _k,\Sigma _k,v_k) \end{aligned}$$

We utilize an Expectation-Maximization (EM) algorithm to iteratively estimate parameters of the SMM. Given a multitude of parameters in the model, a conventional MLE-based EM tends to overfit the data. To mitigate this problem, we introduce priors on the model parameters for model regularization purpose: we use a conjugate Dirichlet prior on $\Pi$ and a normal-inverse Wishart (NIW) prior on $\mu _k$,$\Sigma _k$:

2 $$\begin{aligned} \begin{array}{c} \Pi \thicksim \textrm{Dir}(\Pi |\alpha ^0),\\ \mu _k,\Sigma _k\thicksim NIW(\mu _k,\Sigma _k|m_0,\kappa _0,S_0,\rho _0),\forall k\in [1,K] \end{array} \end{aligned}$$

To simplify the EM algorithm, we rewrite the Student’s t distribution as a Gaussian scale mixture by introducing an “artificial” hidden variable $\zeta _{i,k},\forall i\in [1,N],\forall k\in [1,K]$ that follows a Gamma distribution parameterized by $v_k{:}$

3 $$\begin{aligned} \Phi (\textrm{z}_i|\mu _k,\Sigma _k,v_k)=\int \mathcal {N}\left( z_i|\mu _k,\frac{\Sigma _k}{\zeta _{i,k}}\right) \Gamma \left( \zeta _{i,k}|\frac{v_k}{2},\frac{v_k}{2}\right) d\zeta _{i,k}. \end{aligned}$$

We also introduce a missing variable $\xi _i$ to represent the component membership of z$_i.$ Then the posterior complete data $\log$ likelihood can be written as:

4 $$\begin{aligned} \ell _{c}(\Theta ) & =\mathrm {\log P}(Z,\zeta ,\xi |\Theta )\nonumber \\ & =\sum _i\sum _k\left[ II(\xi _i=k)\left( \log \pi _k+\log \Phi (\textrm{z}_i,\zeta _{i,k}|\mu _k,\Sigma _k,v_k)\right) \right] \nonumber \\ & \quad +\log Dir(\Pi |\alpha ^0)+\sum _k\log NIW(\mu _k,\Sigma _k|m_0,\kappa _0,S_0,\rho _0). \end{aligned}$$

In the *t*-th iteration of the E-step, the expected sufficient statistics $\overline{\xi _{i,k}}^{(t)}$ and $\overline{\zeta _{i,k}}^{(t)}$ are derived based on $\Theta ^{(t-1)}.$ In the subsequent M-step, $\Theta ^{(t-1)}$ is updated to $\Theta ^{(t)}$ by maximizing the auxiliary function $Q(\Theta ,\Theta ^{(t-1)})=E(\ell _c(\Theta )|\Theta ^{(t-1)}).$ Note that $\upsilon _k^{(t)}$ is estimated via a Generalized EM (GEM) technique to speed up the calculation without harming its converging to at least a local optimum. The two steps are alternatively conducted until either convergence is achieved or a pre-specified maximum number of iterations is reached. Refer to Additional file 3: Note 6 for details about the model inference.

### Self-paced pseudo-contrastive optimization of sGRs

Two loss functions, $\mathcal {L}_1$ and $\mathcal {L}_2$, are calculated based on clustering results for updating parameters of both representation learning and the SMM through loss gradient backpropagation. This iterative process progressively improves the clustering-oriented image embeddings and clustering results. Upon completing the inference of SMM parameters $\widetilde{\Theta }$ in each epoch, let *W* and $\widehat{W}$ represent the parameters of the encoder and decoder of the representation learning model respectively, an epoch-level loss $\mathcal {L}_1$ is calculated for updating parameters of MAE :

5 $$\begin{aligned} \mathcal {L}_1=-\mathcal {L}_{\ell \ell }(\textrm{Z}|\Theta )+\eta _1\mathcal {L}_{\ell ap}-\eta _2\mathcal {L}_{size}(\textrm{Z}|\Theta )+\eta _3\mathcal {L}_r(\textrm{Z},\widehat{W}) \end{aligned}$$

Here, $\mathcal {L}_{\ell ap}$ is a Laplacian regularization term that promotes the similarities among image embeddings $\textrm{Z}$ to be consistent with a seeding image-image similarity matrix $\mathcal {S}$, informing the initial training phase. The derivation of $\mathcal {S}$ is detailed in Additional file 3: Note 7. $\mathcal {L}_{\ell ap}$ is defined as follows:

6 $$\begin{aligned} \mathcal {L}_{\ell ap}=Tr\left( Z^{T}\left( I-\mathcal {D}^{-\frac{1}{2}}\mathcal {S}\mathcal {D}^{-\frac{1}{2}}\right) Z\right) , \end{aligned}$$

where $\mathcal {D}$ is the degree matrix of $\mathcal {S}$, and $\eta$, initially set at 0.5, decays over the training course so that the influence of $\mathcal {S}$ is gradually reduced. $\mathcal {L}_{\ell \ell }$ represents the log likelihood of the embeddings given the estimated SMM parameters $\widetilde{\Theta }$:

7 $$\begin{aligned} \mathcal {L}_{\ell \ell }=\sum _{\textrm{i}=1}^{\textrm{N}}\textrm{log}\left[ \sum _{k}q_{i,k}\right] , \end{aligned}$$

8 $$\begin{aligned} q_{i, k} = \pi _{k}\Phi (\textrm{z}_{i}|\mu _{k},\Sigma _{k},v_{k}), \forall i\in [1,\, N], \, \forall k\in [1,\, K]. \end{aligned}$$

$\mathcal {L}_{size}$ penalizes empty and tiny clusters, while exempting those whose size exceeds a predefined threshold $\upsilon$ so that image assignments is not overly uniform:

9 $$\begin{aligned} \mathcal {L}_{\text {size}} = \sum _{k=1}^{K} -J_{k} \log J_{k}, \quad J_k = \left\{ \begin{array}{ll} \frac{\sum \nolimits _{i=1}^{N} q_{i,k}}{N} & \text {if}\ J_k \le \upsilon , \\ 1 & \text {otherwise}. \end{array}\right. \end{aligned}$$

$\mathcal {L}_{r}$, represents the fidelity loss of the reconstructed gene image by the convolutional autodecoder, expressed as:

10 $$\begin{aligned} \mathcal {L}_r = \sum _{i=1}^{N} \Vert x_i - \hat{x}_i\Vert _2^2 = \textrm{Tr}\left( (X - {\hat{X}})(X - {\hat{X}})^\top \right) \end{aligned}$$

where $\hat{x}_i=f_{decoder}(\widehat{W},z_i)$. This term supervises the training of encoder, guiding it to generate gene embeddings that preserves the local structural integrity of spatial gene expressions. We set $\eta _1 = 0.5$, $\eta _2 = 0.1$, $\eta _3 = 0.1$. Note, the value of $\eta _1$ decays as the training progresses so that the impact of the seeding matrix diminishes over the training course.

Subsequently, within the same epoch, we utilize a batch-level loss:

11 $$\begin{aligned} \mathcal {L}_2=\mathcal {L}_c(Z_b,\Theta )+\lambda _1\mathcal {L}_r(Z_b,{\widehat{W}})+\lambda _2\mathcal {L}_{\ell ap}(\mathcal {S}_b,Z_b) \end{aligned}$$

to update MAE and SMM parameters across successive batches. Here, $\mathcal {L}_{r}$ and $\mathcal {L}_{\ell ap}$ remains same as in Eqs. (10) and (6) except being calculated on the batch-level. $\mathcal {L}_{c}$ boosts high-confidence images, incrementally grouping similar instances while separating dissimilar ones:

12 $$\begin{aligned} \mathcal {L}_{c}=KL(\mathcal {P}|Q)=\sum _{i}^{N}\sum _{j}^{K}\mathbb {p}_{i,j}\log \frac{\mathbb {p}_{i,j}}{\mathbb {q}_{i,j}}, \end{aligned}$$

$$\begin{aligned} \text {where}\ \mathbb {q}_{i,k}=\frac{q_{i,k}}{\sum _cq_{i,c}},\mathbb {p}_{i,k}=\frac{\mathbb {q}_{i,k}^{2}/\sum _{i}\mathbb {q}_{i,k}}{\sum _{c}\left( \mathbb {q}_{i,c}^{2}/\sum _{i}\mathbb {q}_{i,c}\right) } \end{aligned}$$

Here, $q_{i,k}$ is same as in Eq. (8), $\mathbb {q}_{i,k}$ represents the probability of assigning *i*-th gene to the *k*-th SMM component, and $\mathbb {p}_{i,k}$ an auxiliary target distribution that boosts up high-confidence images. After this joint optimization, the training progresses to the next epoch, iterating until the end of the training process. The mathematical derivations of gradients of $\mathcal {L}_1$ and $\mathcal {L}_2$ with respect to *W*, $\widehat{W}$ and $\Theta$ are detailed in Additional file 3: Note 8.

### SIGEL-SPS

The section describes a generalized linear model (GLM)-based method, SIGEL-spatial pattern simulator (SIGEL-SPS), for simulating genes with specific spatial expression patterns in a dataset. It uses a negative binomial (NB) distribution to model gene expressions at various spots, incorporating mean and dispersion parameters that relate to the variance and squared coefficient of variation. The model estimates these parameters through regression analysis, predicting spatial expression levels at desired quantiles in specific tissue regions. By ranking and assigning expression levels based on the NB distribution, the method ensures that the simulated genes reflect the spatial structure inherent in the ST dataset, allowing for precise control over the expression pattern of simulated genes across different spatial regions. For further details, see the Additional file 3: Note 9.

### SIGEL-ETC

As shown in Fig. 6A, SIGEL-ETC is a GAN-based model that uses an encoder to transform gene expression matrices into SGRs, which then serve as inputs for a generator. The generator, consisting of an encoder, a decoder, and a memory bank, reconstructs gene expression profiles using an attention mechanism and a continuously updated embedding queue. The discriminator, an MLP-based network, distinguishes between actual and generated gene expressions. The model adjusts the MAE encoder weights in SIGEL’s *Module I* through adversarial and reconstruction losses, which enables the SGRs to adapt to the particular semantics inherent in the FISH-based ST dataset. This finetuning facilitates the generation of those genes uncovered in the FISH-based dataset with optimized fidelity. For additional details, refer to the Additional file 3: Note 10.

### SIGEL-SVG

In our study, we first developed a method that simulates spatially homogeneous genes using observed spatial transcriptomics data, applying either Negative Binomial or Zero-Inflated Negative Binomial distributions. We estimate parameters directly from the data to simulate genes and generate SGRs for both real and simulated genes. These embeddings enable us to calculate spatial variability scores by comparing real genes to their simulated counterparts using scaled cosine dissimilarity. We then rank the genes by these scores to identify those with notable spatial variations. Details provided in the Additional file 3: Note 11.

### SIGEL-ISC

In our study, we employ the SIGEL-ISC method to optimize the analysis of spatial transcriptomics data. This involves constructing a gene identity matrix *G* to distinguish genes across functional groups, and a similarity matrix *S* to assess gene relationships based on SGRs. We then apply Shi-Malik spectral clustering on an adjacency matrix derived from *S* to organize genes into functionally coherent groups. Using SIGEL-SVG, we calculate spatial variability scores for each gene to identify significant spatial expression variations. These scores are used to filter out redundant data, ensuring that only the most informative gene expressions are retained. The processed data is then fed into a graph neural network to generate spot embeddings for clustering, enhancing the clarity and utility of spatial gene expression analysis. For additional details, refer to the Additional file 3: Note 12.

### Experimental settings

Detailed experimental settings for the SIGEL study are extensively documented in the Additional file 3: Note 1. These include the methodologies for identifying groups of spatially co-expressed genes, protocols for enrichment analysis, techniques for cofunction analysis of intra-cluster genes, criteria for evaluating SIGEL-generated gene embeddings, and the metrics used to assess the overall performance of SIGEL.

## Supplementary Information

Additional file 1: Supplementary Figures. This file contains all supplementary figures referenced in the main text, providing additional support for our findings.

Additional file 2: Supplementary Tables. This file includes all supplementary tables that provide detailed data complementing the analyses presented in the main manuscript.

Additional file 3: Supplementary Note. This file contains supplementary notes, including extended methodological details, further discussion on specific results, or any other supporting text-based information.

## Data Availability

The source code for SIGEL, accompanied by a detailed tutorial, is publicly available on GitHub under the MIT license at https://github.com/WLatSunLab/SIGEL [51]. The specific version of the source code used for the analyses in this manuscript is permanently archived in Zenodo with the identifier DOI: 10.5281/zenodo.16867299 [52]. The datasets used in this study can be acquired as: The mouse hippocampus dataset (ssq-mHippo) can be downloaded from Mouse hippocampus dataset (ssq-mHippo) [18, 53]. The human dorsolateral prefrontal cortex datasets (10x-hDLFPC) are available through the *spatialLIBD* R package [54, 55] at http://spatial.libd.org/spatialLIBD. The human breast cancer dataset can be obtained from 10x Genomics’ spatial gene expression dataset page (10x-hBC) [56], https://github.com/almaan/her2st/tree/master (10x-hBC-H1) [57, 58], and https://www.10xgenomics.com/products/xenium-in-situ/preview-dataset-human-breast (Xenium-hBC) [59, 60]. The three human MTG datasets, including two healthy datasets (10x-hMTG-1-1 and 10x-hMTG-18-64) and an AD dataset (10x-hMTG-2-3), are available in the GEO database (GSE220442) [61, 62]. The mouse embryo dataset based on 10x-Visium (10x-mEmb) can be found at GEO database (GSE178636) [63, 64]. The mouse embryo dataset based on SeqFISH (sqf-mEmb) is obtainable at https://crukci.shinyapps.io/SpatialMouseAtlas/ [65, 66]. The detailed descriptions of the datasets can be found in Additional file 2: Table S1. Moreover, we acquire 2,162 human housekeeping genes from the HRT Atlas (https://housekeeping.unicamp.br) [67, 68] and 15 additional housekeeping genes from previous studies [69, 70]. In addition, we obtained 42 AD-associated genes from the literature [71–86].

## Code availability

The code generated in this study is available from the corresponding author upon request.
