# Supplementary material for: SIGEL: a context-aware genomic representation learning framework for spatial genomics analysis
Source: Genome Biol. 2025 Sep 22;26:287. doi: 10.1186/s13059-025-03748-7 (PMC12455800; doi:10.1186/s13059-025-03748-7)
Supplement: Supplementary file 1 — Additional file 1: Supplementary Figures. This file contains all supplementary figures referenced in the main text, providing additional support for our findings. [file 13059_2025_3748_MOESM1_ESM.pdf]

ADDITIONAL FILE 1

SIGEL: a context-aware genomic representation learning framework for spatial genomics analysis

Wenlin Li[1,†], Maocheng Zhu[2], Yucheng Xu[3], Mengqian Huang[2], Ziyi Wang[2], Jing Chen[2], Hao Wu[4,5,*], and Xiaobo Sun[6,†,*]

[1]School of Data Science, The Chinese University of Hong Kong, Shenzhen, Shenzhen, 518172, China

[2]School of Statistics and Mathematics, Zhongnan University of Economics and Law, Wuhan, 430073, China

[3]School of Statistics and Data Science, Nankai University, Tianjin, 300071, China

[4]Faculty of Computer Science and Control Engineering, Shenzhen University of Advanced Technology, Shenzhen, 518055, China

[5]Shenzhen Institute of Advanced Technology, Chinese Academy of Sciences, Shenzhen, 518055, China

[6]Department of Human Genetics, School of Medicine, Emory University, Atlanta, GA, 30322, U.S.

[†]These authors contributed equally: Xiaobo Sun, Wenlin Li

[*]Corresponding authors: xiaobo.sun@emory.edu, wuhao@suat-sz.edu.cn

Supplementary Figures

Fig. S1 Spatial expression of genes in SIGEL-identified co-expression groups.

Fig. S2 SIGEL identifies clusters of spatially co-expressed genes within a biologically relevant genomic context.

Fig. S3 Hierarchical clustering of gene families.

Fig. S4 SGR-based enhancement of transcriptomic coverage of Xenium-based ST with SIGEL-ETC.

Fig. S5 SGR-based SVG detection with SIGEL-SVG on 10x-hDPFC-151507 dataset.

Fig. S6 SGR-based SVG detection with SIGEL-SVG on 10x-hBC dataset.

Fig. S7 SGR-based spatial clustering with SIGEL-SC.

Fig. S8 Computational efficiency and sensitivity analysis of SIGEL.

Fig. S1 Spatial expression of genes in SIGEL-identified co-expression groups.

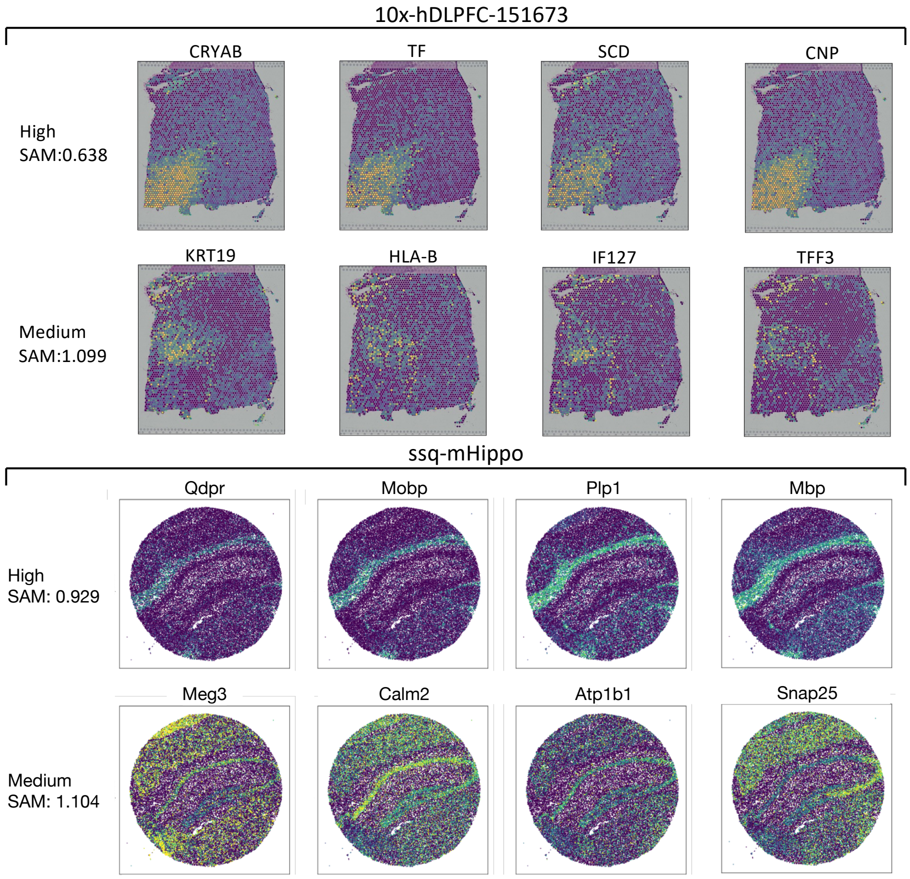

Spatial expression patterns of individual genes within co-expression groups identified by SIGEL from the human DLPFC 10x Visium (10x-hDLPFC-151673) and the mouse hippocampus Slide-seqV2 (ssq-mHippo) datasets, respectively. For each dataset, the denoised expression patterns of four genes randomly selected from the same group are shown in the same row. The first and second rows represent two gene clusters of high and medium intra-cluster similarity respectively, as assessed by the SAM metric. A lower SAM value indicates higher intra-cluster similarity.

Fig. S2 SIGEL identifies clusters of spatially co-expressed genes within a biologically relevant genomic context.

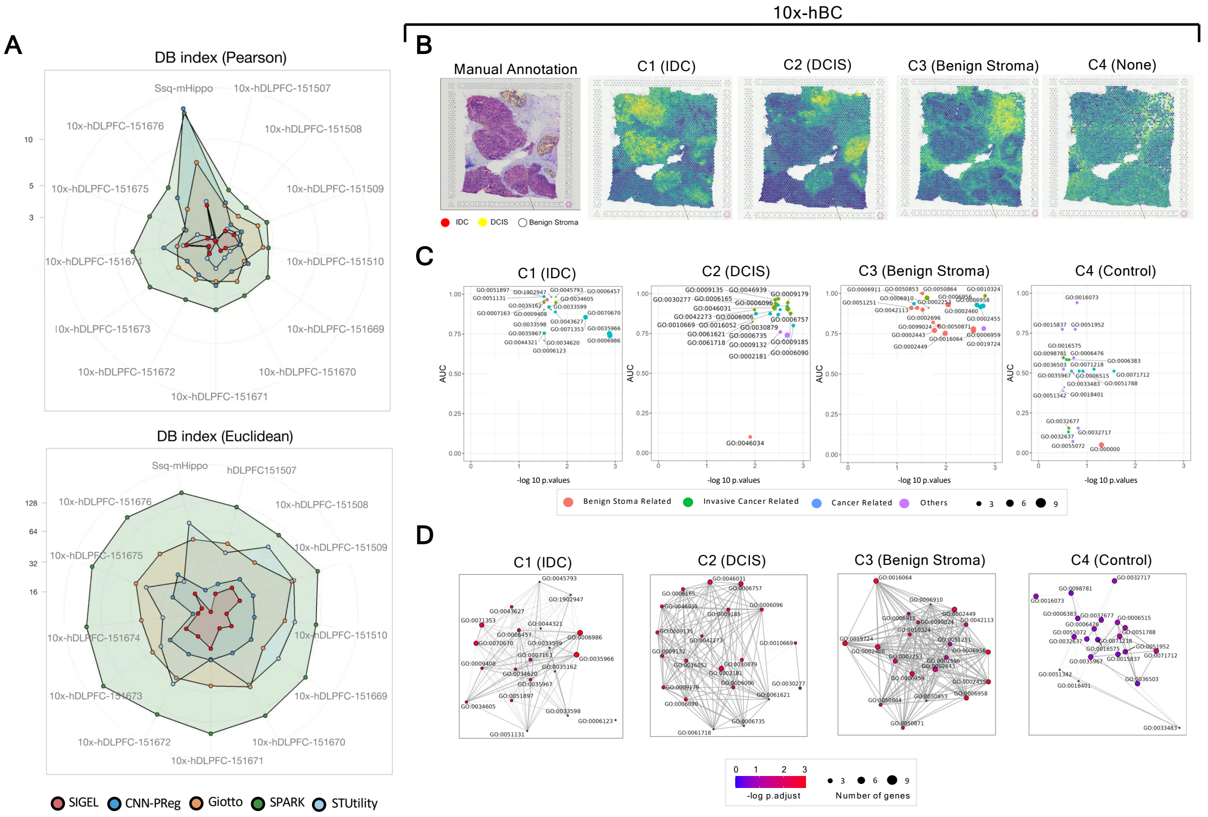

(**A**) Performance comparison of SIGEL and four benchmark methods in grouping co-expressed genes in the 10x-hDLPFC-151673 and ssq-mHippo datasets. The DB index calculated based on Pearson and Euclidean distance are used to measure the overall co-expression (top panel) and spatial coherence (bottom panel) of the gene clusters, respectively. In both cases, a lower DB index value indicates enhanced co-expression or spatial coherence. (**B**) Spatial expression patterns of SIGEL-generated gene groups overlap with the cell type distributions in the 10x-hBC dataset. The leftmost panel displays the manually annotated distributions of ductal carcinoma in situ (DCIS, in yellow), invasive ductal carcinoma (IDC, in red) and benign stroma cells (in original color) in human breast cancer tissues. The right four panels display the aggregated spatial expression (module scores) of three SIGEL-generated gene groups (C1-C3) and a control cluster (C4) consisting of randomly selected genes. The brightness in each panel is positively correlated with the level of aggregated gene expression. The name of the cell type, whose distribution overlaps with the aggregated expression pattern of the gene group, are indicated above each panel. (**C**) GO enrichment and cofunction analyses of genes within C1-C4. The dots represent the 20 most significantly enriched GOBPs in each gene group. The x-axis represents the negative logarithm of adjusted P-values of biological process enrichment significance, while the y-axis represents the ROC AUC scores of the 20 GOBPs in the gene cofunction analysis. Red color indicates benign stroma-related GOBPs, green color the noninvasive cancer-related GOBPs, cyan color invasive cancer-related GOBPs, and purple color other GOBPs. (**D**) The connectivity between the nodes corresponding to most significantly enriched GOBP indicates their functional associations. The node color represents the GOBP's enrichment significance (negative logarithm of adjusted P-value), with darker colors indicative of lower significance levels. Node size indicates the number of genes involved in the GOBP.

Fig. S3 Hierarchical clustering of gene families.

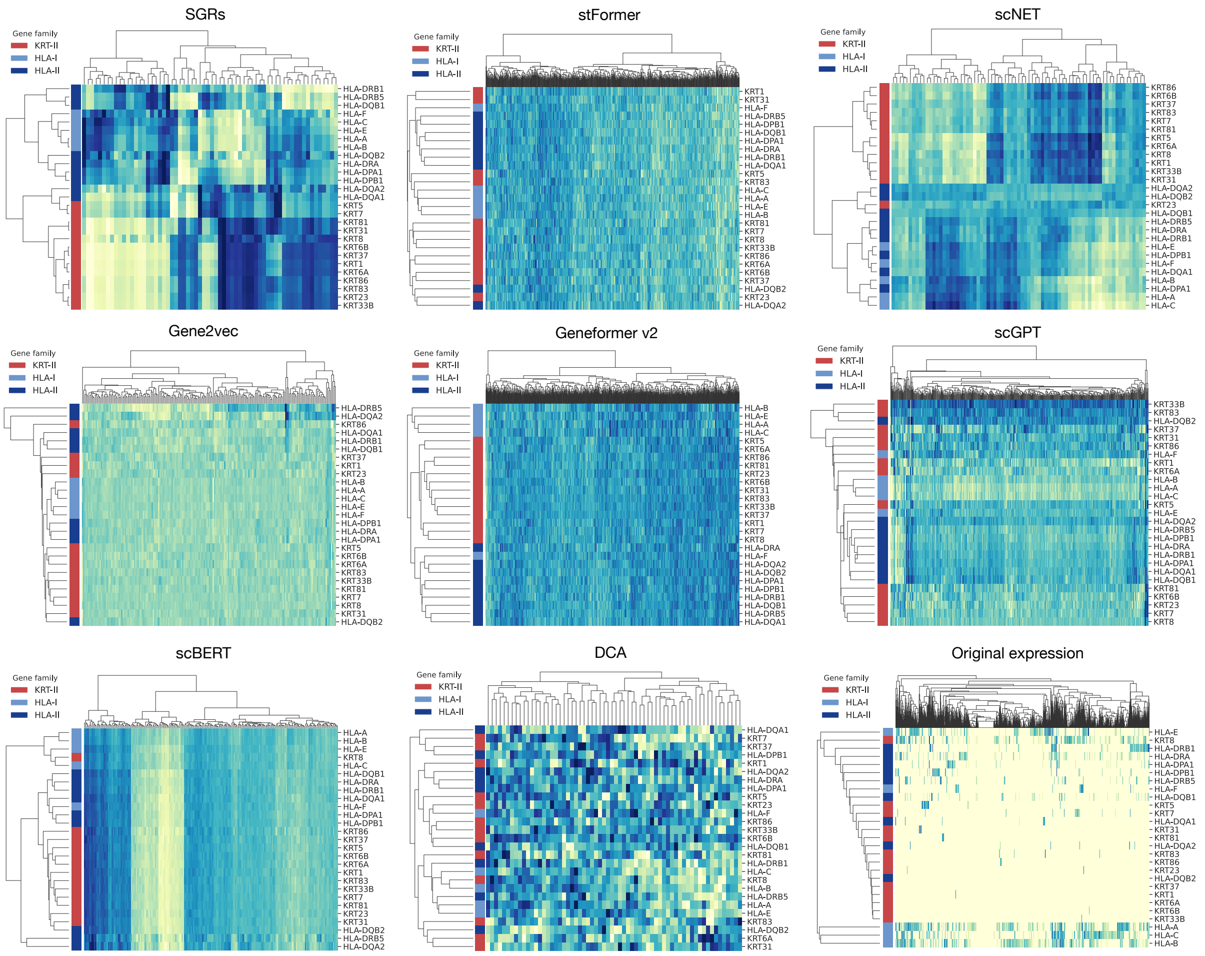

Hierarchical clustering of HLA-I, HLA-II, and KRT-II gene family members using different gene representations, including SGRs, stFormer, scNET, Gene2Vec, Geneformer v2, scGPT, scBERT, DCA-generated embeddings, and original gene expression. The y-axis displays gene family members, ordered by hierarchical clustering of SGRs. Gene families are indicated by colors on the y-axis. The x-axis displays the dimensions of the gene representations (embeddings or original gene features). Blue color intensity positively correlates with the corresponding gene representation values.

Fig. S4 SGR-based enhancement of transcriptomic coverage of Xenium-based ST with SIGEL-ETC.

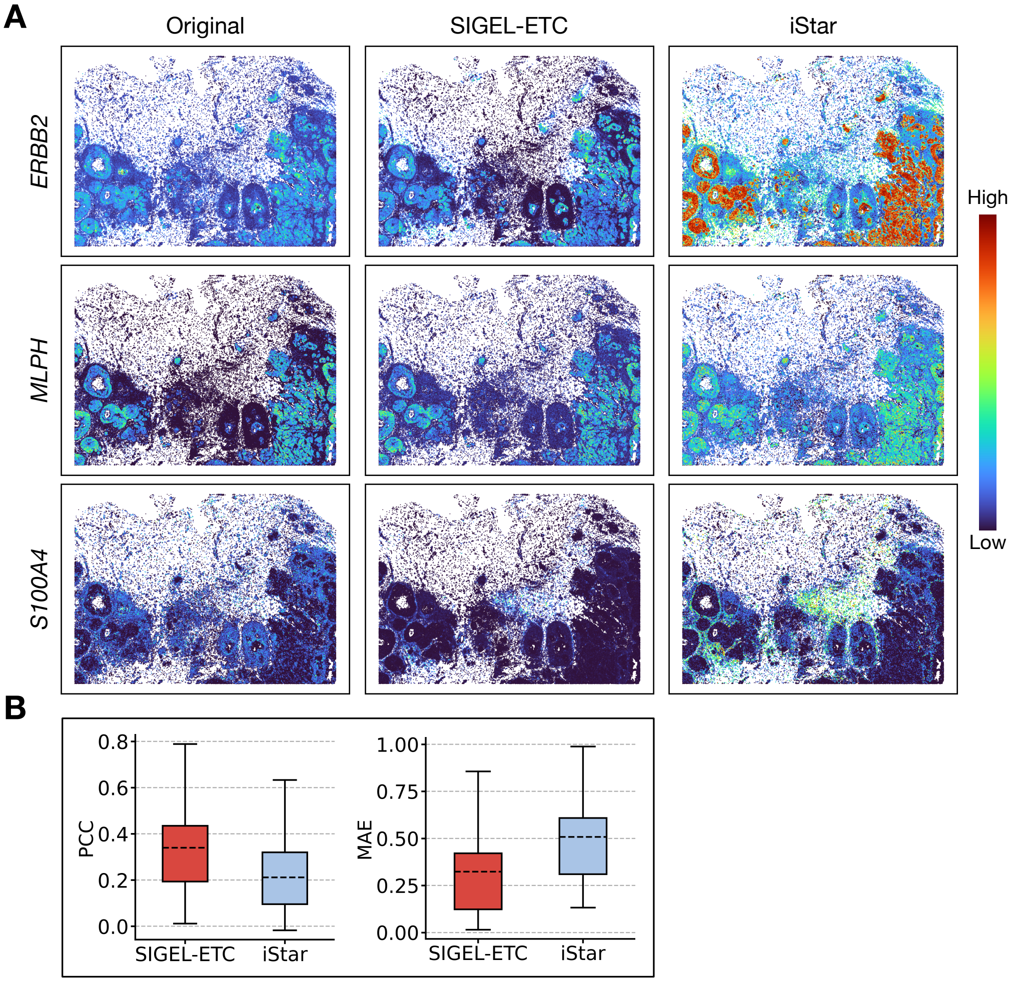

(**A**) Three genes (*ERBB2*, *MLPH*, and *S100A4*), which are covered by both the 10x-hBC-H1 and the Xenium-hBC datasets and differ in their expression levels, are used to evaluate SIGEL-ETC. From the left to the right, we show the three genes' original spatial expression profiles in the Xenium-hBC dataset, alongside their regenerated expression profiles by SIGEL-ETC and iStar. (**B**) Displayed in the box plot are the Pearson correlation coefficients and mean absolute errors between the original and spatial expression profiles of the 150 genes regenerated by SIGEL-ETC and iStar.

Fig. S5 SGR-based SVG detection with SIGEL-SVG on 10x-hDPFC-151507 dataset.

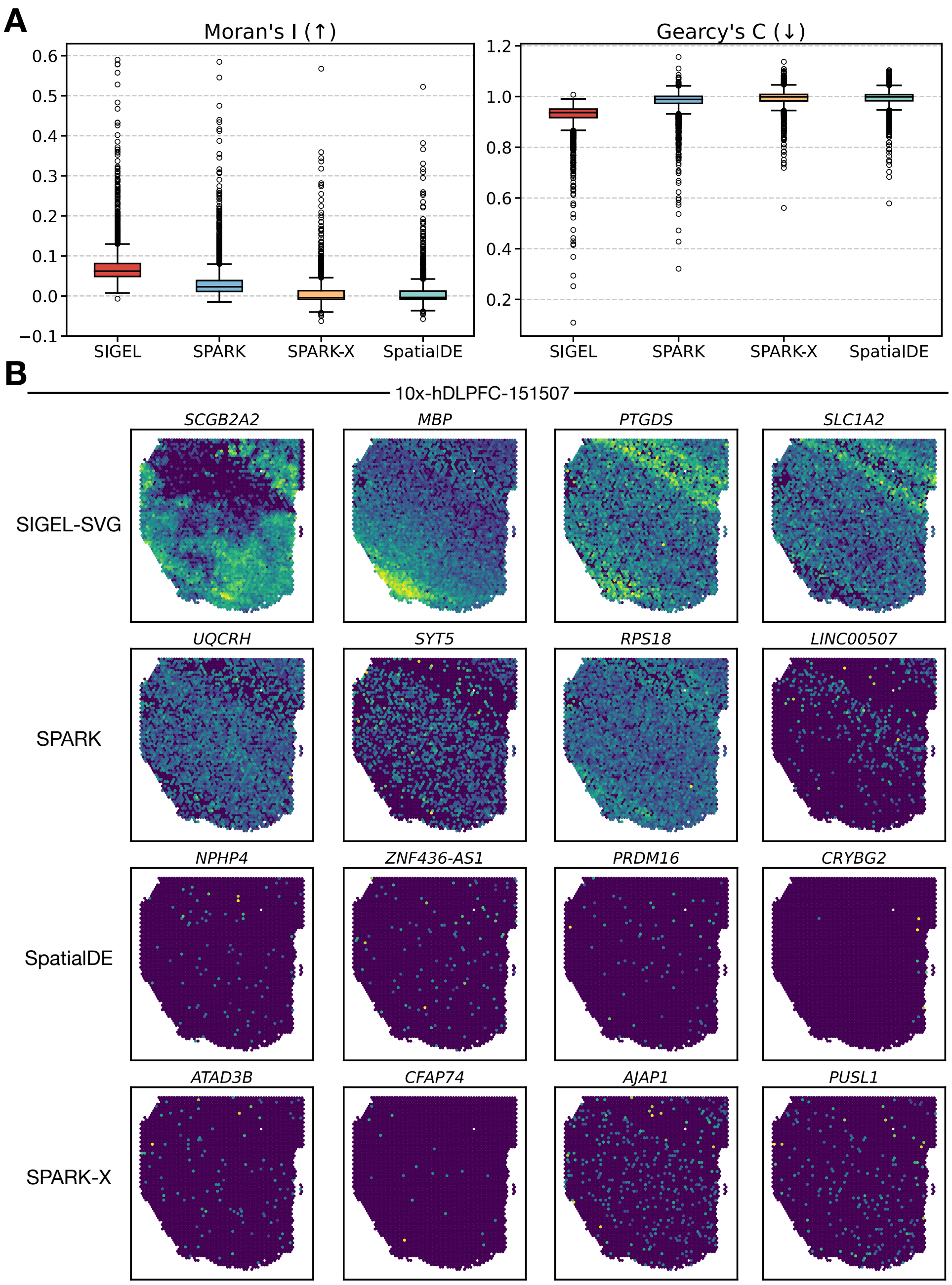

(**A**) The box plots show the Moran's I (left panel) and Geary's C (right panel) indices for the top 3000 SVGs detected by SIGEL-SVG, SPARK, SPARK-X and SpatialDE from the 10x-hDLPFC-151507 dataset. The value ranges for these indices are [-1,1] for Moran's I and [0, 2] for Geary's C. A value approaching the upper limit for Moran's I (or the lower limit for Geary's C) indicate a stronger positive autocorrelation and a more pronounced spatial pattern. (**B**) The spatial expression patterns of the top four SVGs identified by SIGEL-SVG, SPARK, SPARK-X, and SpatialDE are shown in four rows, each corresponding to one method from top to bottom. The panels in the top row have the clear spatial patterns, while those in the other three rows are almost devoid of noticeable spatial patterns.

Fig. S6 SGR-based SVG detection with SIGEL-SVG on 10x-hBC dataset.

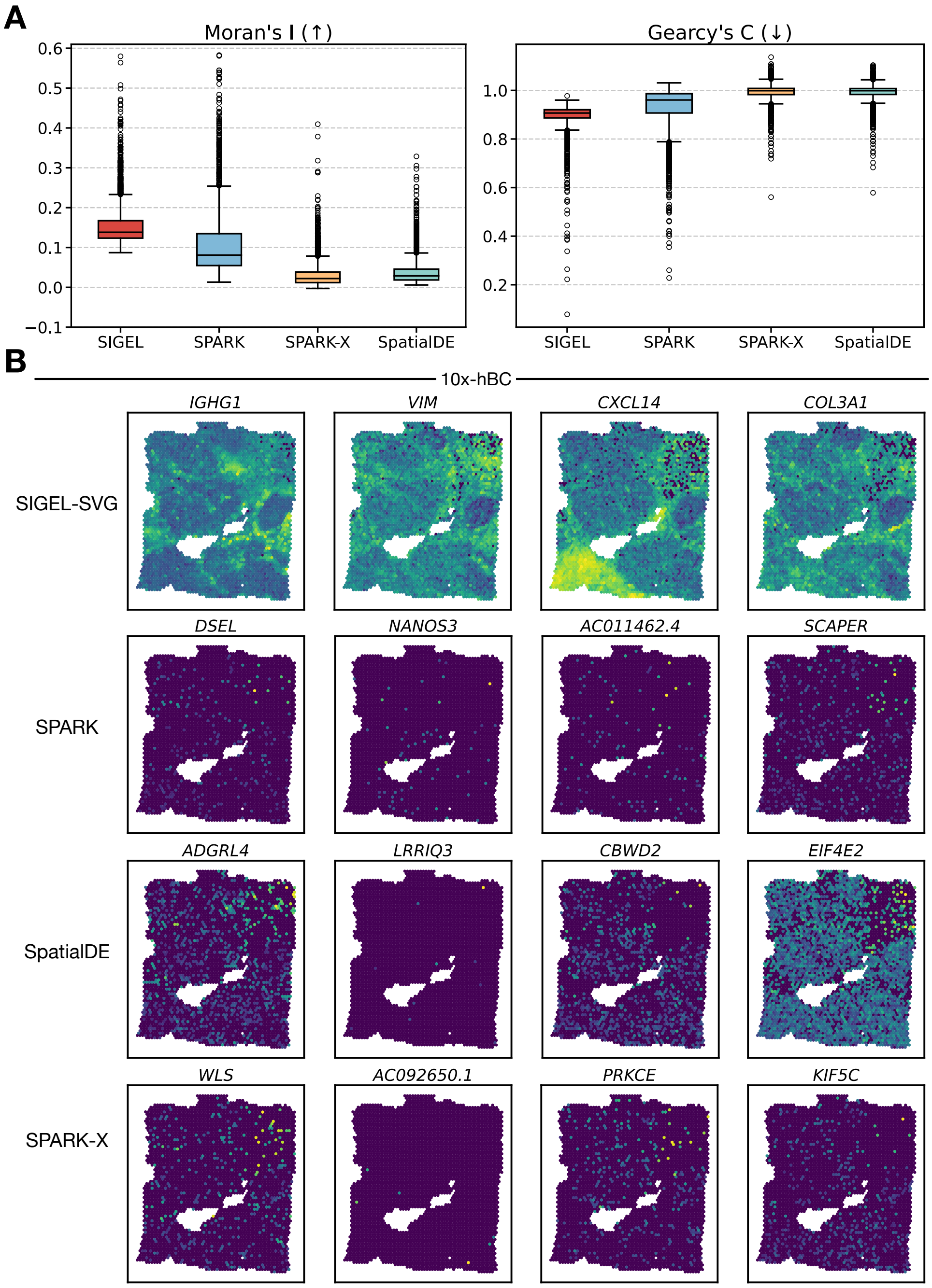

(**A**)The box plots present Moran's I (left) and Geary's C (right) indices for the top 3000 SVGs identified by SIGEL-SVG, SPARK, SPARK-X, and SpatialDE on the 10x-hBC dataset. Moran's I ranges from −1 to 1, while Geary's C ranges from 0 to 2. Higher Moran's I or lower Geary's C values indicate stronger positive spatial autocorrelation and more distinct spatial expression patterns. (**B**) The top four SVGs identified by SIGEL-SVG, SPARK, SPARK-X, and SpatialDE are shown from top to bottom, with each row corresponding to one method. The top row exhibits clear spatial expression patterns, whereas the remaining rows show little to no discernible spatial structure.

Fig. S7 SGR-based spatial clustering with SIGEL-SC.

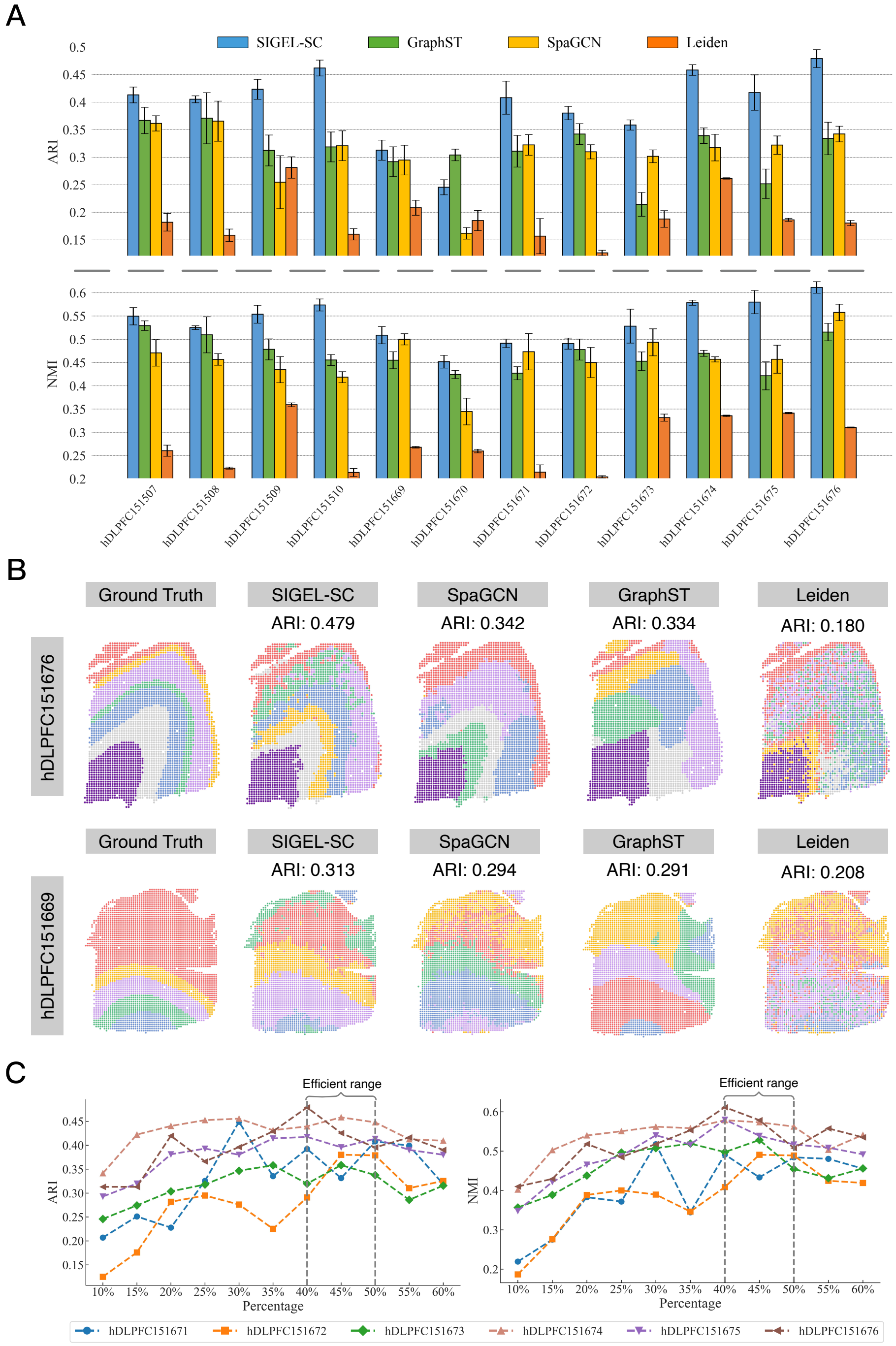

(**A**) The bar plots illustrate the accuracy of spatial clustering across twelve 10x-hDLPFC datasets using SIGEL-SC, GraphST, SpaGCN and Leiden in terms of ARI in the first row and NMI in the second row. (**B**) We randomly select two datasets (10x-hDLPFC-151676 and 10x-hDLPFC-151669) to visualize their ground truth domain annotations and spatial clustering results across methods. (**C**) The line plots showcase the trend of spatial clustering accuracies with percentages of SIGEL-SC-filtered redundant gene information across the six 10x-hDLPFC datasets. The spatial clustering performances peaks when approximately 50%-60% redundant information is excluded.

Fig. S8 Computational efficiency and sensitivity analysis of SIGEL.

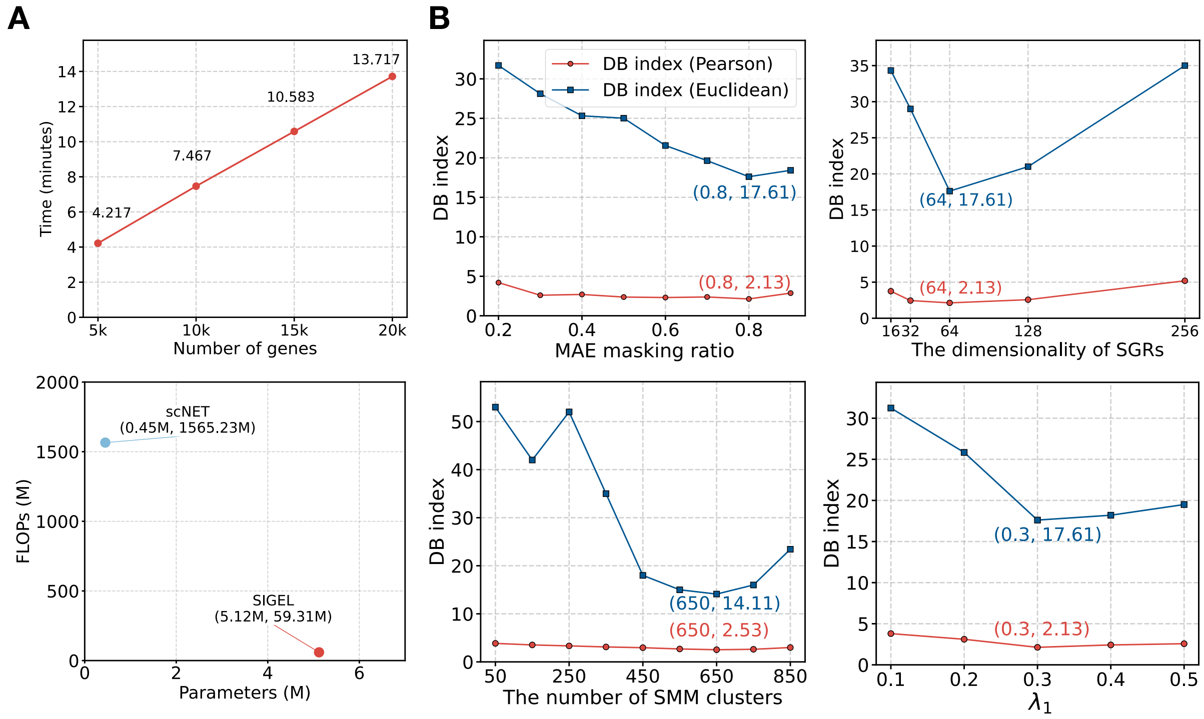

(**A**) The top panel shows SIGEL's training time (in minutes) as a function of the number of genes on the hDLPFC-151676 dataset. The bottom panel compares the number of parameters and FLOPs among SIGEL and scNET on the same dataset. (**B**) Sensitivity analysis of SIGEL's clustering performance with respect to four hyperparameters: mask ratio, SGR dimensions, SMM cluster number, and the weight η_1 of the reconstruction loss in $\mathcal{L}_2$. Clustering performance was evaluated using two variants of the Davies–Bouldin (DB) index: one based on Pearson correlation and the other on Euclidean distance. In both cases, lower DB index values indicate better clustering quality.
