# Supplementary material for: SIGEL: a context-aware genomic representation learning framework for spatial genomics analysis
Source: Genome Biol. 2025 Sep 22;26:287. doi: 10.1186/s13059-025-03748-7 (PMC12455800; doi:10.1186/s13059-025-03748-7)
Supplement: Supplementary file 3 — Additional file 3: Supplementary Note. This file contains supplementary notes, including extended methodological details, further discussion on specific results, or any other supporting text-based information. [file 13059_2025_3748_MOESM3_ESM.pdf]

ADDITIONAL FILE 3

SIGEL: a context-aware genomic representation learning framework for spatial genomics analysis

Wenlin Li[1,†], Maocheng Zhu[2], Yucheng Xu[3], Mengqian Huang[2], Ziyi Wang[2], Jing Chen[2], Hao Wu[4,5,*], and Xiaobo Sun[6,†,*]

[1]School of Data Science, The Chinese University of Hong Kong, Shenzhen, Shenzhen, 518172, China

[2]School of Statistics and Mathematics, Zhongnan University of Economics and Law, Wuhan, 430073, China

[3]School of Statistics and Data Science, Nankai University, Tianjin, 300071, China

[4]Faculty of Computer Science and Control Engineering, Shenzhen University of Advanced Technology, Shenzhen, 518055, China

[5]Shenzhen Institute of Advanced Technology, Chinese Academy of Sciences, Shenzhen, 518055, China

[6]Department of Human Genetics, School of Medicine, Emory University, Atlanta, GA, 30322, U.S.

[†]These authors contributed equally: Xiaobo Sun, Wenlin Li

[*]Corresponding authors: xiaobo.sun@emory.edu, wuhao@suat-sz.edu.cn

Supplementary Notes

Note 1 Experimental settings

- **Note 1.1** Identifying groups of spatially co-expressed genes
- **Note 1.2** Evaluation Metrics
- **Note 1.3** Evaluating SIGEL-generated gene embeddings
- **Note 1.4** Cofunction analysis of intra-cluster genes
- **Note 1.5** Enrichment analysis

Note 2 Calculating Group Closeness Centrality of Gene Clusters

Note 3 Visualization of the Aggregated Gene Expression Pattern of a Gene Cluster

Note 4 Biological Analysis of SIGEL-identified Clusters

Note 5 Predicting gene–gene interactions using embeddings

Note 6 MAP-EM Inference of SMM Parameters

Note 7 Deriving the Seeding Gene–Gene Similarity Matrix

Note 8 The Joint Optimization of the Gene Representation Learning and SMM via a Discriminative Boosted Clustering

Note 9 SIGEL-SPS

- **Note 9.1** Fisher Scoring Method for Estimating GLM Parameters

Note 10 SIGEL-ETC

Note 11 SIGEL-SVG

Note 12 SIGEL-ISC

Note 13 Supplementary Algorithm

Note 1 Experimental settings

Note 1.1 Identifying groups of spatially co-expressed genes

Two important settings in this experiment include the target number of clusters and how to choose gene clusters for visualizing the spatial expression patterns of their member genes. The target number of gene clusters is based on the total number of genes in the *dataset*, aiming for an average of ~30 genes per cluster to approximate the average number of genes in a typical KEGG pathway [46]. For the second setting, gene clusters are first categorized into three groups according to their *intra-cluster* gene similarity quantified by averaging the spatial *autocorrelation* measure (SAM, see "*Evaluation metrics*" in *Methods*) values among *intra-cluster* genes: the high-quality group (top third *quantile*), the low-quality group (bottom third *quantile*), and the medium-quality group (all remaining clusters). Subsequently, one cluster is randomly selected for visualization from each of the high- and medium-quality groups of the 10x-hDLFPC-151673 and ssq-mHippo datasets.

Note 1.2 Evaluation Metrics

Gene clustering. Davies-Bouldin (DB) index is used to assess the performance of gene clustering:

$$\mathrm{DB} = \frac{1}{n}\sum_{i=1}^{n}\max_{i\neq j}\left(\frac{d_i + d_j}{d_{(i,j)}}\right) \tag{1}$$

$$d_i = \frac{2}{n_i(n_i - 1)}\sum_{p,q\in G_i, p\neq q}\delta_{p,q} \tag{2}$$

$$d_{(i,j)} = \delta_{c_i,c_j} \tag{3}$$

where $\delta_{p,q}$ denotes the distance between genes p and q, n denotes the number of clusters, d_i is the intra-cluster distance of cluster i (calculated as the average distance between genes of cluster i), and $d_{(i,j)}$ is the inter-cluster distance between clusters i and j (calculated as the distance between cluster centroids c_i and c_j). DB index represents the ratio of intra-cluster distance (compactness) to inter-cluster distance (separateness), so a smaller value of the DB index indicates better clustering performance. To evaluate the co-expression and the spatial coherence of gene clusters, Pearson distance and Euclidean distance are used in the calculation of the DB index, respectively:

$$\delta_{p,q}^{\text{pearson}} = 1 - \rho_{p,q} \tag{4}$$

$$\delta_{p,q}^{\text{euclid}} = \sqrt{(x_p - x_q)^T W (x_p - x_q)}, \quad W \in \mathbb{R}^{N_x N_y \times N_x N_y} \tag{5}$$

where $\rho_{p,q}$ represents the Pearson correlation between genes p and q. N_x and N_y denote the number of spatial spots along the horizontal and vertical axes of the spatial map,

respectively. x_p is the flattened expression matrix of gene p, and W is the weight matrix calculated based on the spatial spots' locations using a Gaussian kernel, reflecting the spatial closeness of the spatial spots.

SVG detection. Moran's I and Geary's C, which measure the spatial autocorrelation of a variable from different perspectives, are used to evaluate the spatial variability in gene expressions. The two metrics calculated on a gene x are as follows:

$$\text{Moran's I} = \frac{n}{w} \frac{\sum_{i=1}^{n} \sum_{j=1}^{n} \left[w_{ij} (x_i - \bar{x})(x_j - \bar{x}) \right]}{\sum_{i=1}^{n} (x_i - \bar{x})^2} \tag{6}$$

$$\text{Geary's C} = \frac{n}{2w} \frac{\sum_{i=1}^{n} \sum_{j=1}^{n} \left[w_{ij} \left(x_i - x_j \right)^2 \right]}{\sum_{i=1}^{n} (x_i - \bar{x})^2} \tag{7}$$

$$w_{ij} = \begin{cases} 1, & \text{if } j \in N_k(i) \\ 0, & \text{otherwise} \end{cases}$$

$$w = \sum_{i=1}^{n} \sum_{j=1}^{n} w_{ij} \tag{8}$$

where x_i denotes the read counts at spot i, $\bar{x}$ is the average read counts over all spots, n is the total number of spots, w_{ij} is the spatial weight between spots i and j, and $N_k(i)$ is the set of k-nearest neighbors of spot i in Euclidean distance. Moran's I ranges from $[-1, 1]$ and Geary's C ranges from $[0, 2]$. Values closer to the upper boundary for Moran's I (or the lower boundary for Geary's C) indicate stronger positive autocorrelation and a more regular spatial pattern.

Intra-cluster gene similarity. Given any pair of genes within a cluster, the SAM metric, originally designed for measuring imagery similarity, is used to measure the similarity of their spatial expression patterns. Let $\mathbf{x}, \mathbf{y} \in \mathbb{R}^n$ denote the two genes' spatial expression vectors, respectively. Their SAM value is computed as:

$$\text{SAM}(\mathbf{x}, \mathbf{y}) = \cos^{-1} \left(\frac{\sum_{i=1}^{n} x_i \cdot y_i}{\|\mathbf{x}\| \cdot \|\mathbf{y}\|} \right), \tag{9}$$

where n denotes the number of spots.

Then, the intra-cluster gene similarity of cluster c_k is calculated as:

$$\text{SAM}(c_k) = \frac{\sum_{i,j \in c_k, i \neq j} \text{SAM}(\mathbf{x}_i, \mathbf{x}_j)}{|c_k|}, \tag{10}$$

where $|c_k|$ represents the number of gene pairs in c_k.

Spatial clustering. The accuracy of clustering for spatial spots is assessed using the Adjusted Rand Index (ARI) and Normalized Mutual Information (NMI). For ARI, let n denote the total number of spots, n_{ij} the number of spots of domain type i in cluster j,

a_i the total number of spots of domain type i, and b_j the number of all spots in cluster j. The ARI is defined as:

$$\mathrm{ARI} = \frac{\sum_{i,j}\binom{n_{ij}}{2} - \frac{\sum_i\binom{a_i}{2}\sum_j\binom{b_j}{2}}{\binom{n}{2}}}{\frac{1}{2}\left(\sum_i\binom{a_i}{2} + \sum_j\binom{b_j}{2}\right) - \frac{\sum_i\binom{a_i}{2}\sum_j\binom{b_j}{2}}{\binom{n}{2}}}. \quad (11)$$

For NMI, let L denote the true labels of spots, $\tilde{L}$ the cluster labels of spots, MI the mutual information function, and H the entropy function. The NMI is defined as:

$$\mathrm{NMI} = \frac{2 \times \mathrm{MI}(\tilde{L}, L)}{H(\tilde{L}) + H(L)}. \quad (12)$$

Note 1.3 Evaluating SIGEL-generated gene embeddings

This study comprises four analyses aimed at evaluating the relational semantics of SGRs and their utility in gene function ontology-related tasks. For comparison purpose, seven baseline gene embeddings generated by stFormer, scNET, Gene2Vec, scGPT, scBERT, Geneformer v2, and DCA are also included. To ensure a fair comparison, all embeddings are derived from the 10x-hDLFPC-151676 dataset In the first analysis, we collected nine types of gene embeddings (SGRs, seven baseline embeddings, and raw gene expression vectors) for 26 genes from three gene families—*KRT-II*, *HLA-I* and *HLA-II* (see Additional file2: Table S4). We then performed average-linkage hierarchical clustering on these embeddings to group genes with similar representations closer in the dendrogram. In the second analysis, we constructed gene–gene similarity matrices using each embedding type and applied Leiden clustering across a range of resolutions, targeting between 20 and 80 clusters. For each clustering result, we performed pathway enrichment analysis using the *Reactome* database and computed the percentage of clusters enriched for at least one biological pathway. A higher percentage indicates that the resulting gene clusters exhibit greater biological coherence, as genes grouped within the same cluster are more likely to participate in shared biological processes. This, in turn, reflects a stronger capacity of the corresponding gene embeddings to capture biologically meaningful gene relationships. The third analysis assessed the ability of gene embeddings in reflecting GO-based gene functional similarity. Specifically, we computed the GO Resnik similarity for each gene pair [25, 28], which quantifies semantic similarity between GO terms based on the location of their most informative common ancestor in the GO hierarchy. These GO-based similarities were then compared to cosine similarities computed using SGRs and the seven benchmark gene embeddings. The correlation between the two similarity measures indicates how effectively each embedding method encodes GO-defined functional relationships among genes. In the fourth analysis, we trained an enhanced GGIPNN model—a multilayer perceptron classifier with residual connections—to pre-

dict gene–gene interactions using different types of gene embeddings as input. Details on the construction of positive (interacting) and negative (non-interacting) gene pairs are provided in Note 5. This analysis involved nine embedding types, including SGRs, the seven baselines, and randomly generated 64-dimensional embeddings used as a control.

Note 1.4 Cofunction analysis of intra-cluster genes

In this study, we utilize the neighbor-voting method to assess the functional coherence of a gene set within a GOBP. Basically, under the assumption that genes within the same cluster are more functionally coherent and given the GOBP involvement of some genes in a gene cluster, it should yield more accurate prediction on the GOBP involvement of other genes in the same cluster. Specifically, let d denote the number of genes, q denote the number of GOBPs/pathways, and $A \in \mathbb{R}^{d\times q}$ denote the gene annotation matrix. Here,

$$A_{i,j} = \begin{cases} 1, & \text{if gene } i \in \text{ the } j\text{-th GOBP} \\ 0, & \text{otherwise} \end{cases} \tag{13}$$

Next, the scaled cosine gene-gene similarity matrix $S \in \mathbb{R}^{d\times d}$ is calculated based on the gene embeddings $Z = \{z_i, \forall i \in [1, d]\}$ generated by SIGEL:

$$S_{i,j} = \frac{z_i^T z_j}{\gamma \|z_i\| \|z_j\|}, \tag{14}$$

where γ is a temperature hyperparameter. The accuracy of GOBP predictions is evaluated using a three-fold cross-validation approach. In each fold, a random selection of one-third of the genes serves as the testing dataset, with their associated GOBPs masked by setting the corresponding rows in A to zero. For a gene t in the testing dataset, the probability of its involvement in the j-th GOBP is calculated as:

$$p_{tj} = \frac{\sum_{i=1}^{d} S_{t,i} A_{i,j}}{\sum_{i=1}^{d} S_{t,i}} \tag{15}$$

The prediction results are evaluated by examining the top 20 enriched GOBPs, using AUC metric. A higher AUC value indicates greater functional coherence among the tested genes for that GOBP. Genes involved in the selected GOBPs are obtained using R package `AnnotationDbi` (v1.56.2). The R package `EGAD` (v1.22.0) is employed to conduct the neighbor-voting-based prediction.

Note 1.5 Enrichment analysis

Enrichment analysis is employed to identify biological functions or processes enriched in the investigated gene set. Here, we conduct GO pathway enrichment analyses on gene groups to assess their biological relevance to breast cancer. Among the three data types

in the GO database (GOBPs, molecular functions and cellular components), we only consider GOBPs since they better reflect high-level functions of the investigated system. The R package `clusterProfiler` (v4.2.2) is used to identify GOBPs pathways enriched in the genes of the two groups. In each enrichment analysis, we report P-values with false discovery control adjusted by Benjamini-Hochberg procedure. The 20 most significantly enriched GOBPs/KEGG pathways are selected for further biological analyses.

Note 2 Calculating Group Closeness Centrality of Gene Clusters

The group closeness centrality is a metric originally developed for quantifying the "closeness" of a group of nodes to other nodes on a graph. It can be used to measure the aggregated expression similarity between a gene cluster and other gene clusters on a weighted gene graph, with nodes representing genes and edge weights representing the expression dissimilarity between a pair of genes.

In this study, we first convert the gene-to-gene similarity matrix $S \in \mathbb{R}^{d \times d}$ (see "Co-function Analysis of Intra-cluster Genes" in Note 1.4) into a distance matrix $D \in \mathbb{R}^{d \times d}$:

$$D = M \times \mathrm{Diag}(S) + \mathrm{Diag}(S) \times M - 2S,$$

where $M \in \mathbb{R}^{d \times d}$ is a matrix full of 1s. Based on D, the group closeness centrality of a gene cluster G, $\mathrm{gcc}(G)$, is calculated as:

$$\mathrm{gcc}(G) = \frac{|V - G|}{\sum_{v \in \{V-G\}} d_{G,v}},$$

where $d_{G,v} = \min_{u \in G}(D_{u,v})$, V represents the entire set of gene nodes, and $|V - G|$ represents the number of gene nodes not belonging to G.

Note 3 Visualization of the Aggregated Gene Expression Pattern of a Gene Cluster

To depict the collective gene expression pattern of a target gene cluster on the spatial map, we employ the `AddModuleScore` function from the Seurat R package to calculate module scores for the cluster across all spatial spots [23]. Specifically, we bin all genes based on their average expression. Then a number of control genes are randomly selected from each bin to match the number of genes of the target gene cluster that reside in the same bin. Consequently, selected control genes should have a similar expression distribution to the genes within the target cluster but be functionally unrelated.

The differences between the average expressions of the set of cluster genes and the set of control genes serve as the cluster's module scores across all spatial spots. Finally, a color spectrum is utilized to display module scores on the spatial map, with brighter hues signifying higher module scores, and vice versa.

Note 4 Biological Analysis of SIGEL-identified Co-expressed Gene Clusters

To explore the biological processes associated with C1–C4, we performed GO enrichment analysis on their member genes and identified the top 20 most significantly enriched Gene Ontology Biological Processes (GOBPs) for each cluster. As shown in Additional file 1: Fig. S2C, all clusters except C4 exhibited statistically significant GOBPs that form densely interconnected functional networks. Notably, both C1 (17 GOBPs) and C2 (18 GOBPs) were enriched in cancer-related biological processes (Additional file 2: Table S3). Interestingly, C1 showed more enrichment in invasive cancer-related processes than C2, consistent with the observation that genes in C1 are highly expressed in regions rich in invasive ductal carcinoma (IDC). In contrast, C3 was enriched in fewer cancer-related GOBPs but more benign stroma-associated processes. For example, member genes such as COL6A2 and COL1A2 in C3 are involved in forming the extracellular matrix of benign stromal tissue. Meanwhile, C4 showed no significant enrichment in any GOBP. To further assess the functional coherence within each cluster, we evaluated whether the involvement of a gene in a significantly enriched GOBP could be predicted based on the involvement of other member genes in the same GOBP (see "Cofunction Analysis of Intra-cluster Genes" in Note 1.4). Prediction accuracy was quantified using the area under the ROC curve (AUC). Results demonstrated that C1 through C3 achieved higher predictive performance than C4, as indicated by their higher AUC values (Additional file1: Fig. S2C).These findings suggest that SIGEL is capable of identifying gene clusters with strong cofunctional coherence and biological or pathological relevance to the studied tissue context.

Note 5 Predicting gene-gene interactions using gene embeddings

In this analysis, we adopt the methodology proposed by Du et al. [1]. A pair of genes is considered interacting (*positive*) if they share GO terms obtained using the R package "org.Hs.eg.db"; otherwise, they are considered noninteracting (*negative*). To reduce the number of false positive gene pairs, we omit the highly over-represented GO terms, including "single transduction" (GO:0007165), three terms related to phosphorylation ("protein amino acid phosphorylation", GO:0006468; "protein amino acid autophosphorylation", GO:0046777; "protein amino acid dephosphorylation", GO:0006470), as well as all terms in the first three layers of the GO hierarchy.

The universal gene set for this experiment is defined by the intersection of genes present in the hDLPFC dataset and the GO database, resulting in a total of 14,618 genes. Within this set, there are 778,293 positive samples involving 8,600 human genes and 37,577,368 negative samples involving 8,759 human genes. All positive samples are included in the positive dataset, and an equal number of negative samples are randomly selected to constitute the negative dataset. Subsequently, 80% of gene pairs are sampled from both the positive and negative datasets to create the training dataset, while the remaining 20% constitute the testing dataset.

A gene-gene interaction predictor neural network (GGIPNN) [1], which takes gene embeddings as inputs, is employed to predict gene-gene interactions. **Main Text, Fig. 3D** illustrates that the GGIPNN, when using SGRs, achieves higher accuracy (0.779 ± 0.0048) and AUC (0.831) scores than those using scBERT gene embeddings (accuracy $= 0.742 \pm 0.0052$, AUC = 0.812), Gene2vec gene embeddings (accuracy $= 0.726 \pm 0.0026$, AUC = 0.794), and random embeddings (accuracy $= 0.500 \pm 0.0014$, AUC = 0.510).

Additionally, we introduce a bicolor heatmap to visualize the interactions among the top 1,000 most interactive genes, with filled cells denoting gene interactions. **Main Text, Fig. 3E** showcases that the heatmap of predictions with SGRs exhibits greater concordance with the ground truth than those with other embeddings.

Note 6 MAP-EM Inference of SMM Parameters

We first list the mathematical notations used in the inference below:

$X \in \mathbb{R}^{N\times M}$: the raw gene count matrix.
N: the number of genes.
M: the number of spatial spots.
K: the number of gene clusters (components in the SMM).
$\mathbf{Z} \in \mathbb{R}^{N\times D} = f_{\text{encoder}}(W, X)$: the SIGEL-generated gene embeddings.
W: parameters of the MAE encoder.
$\hat{\mathbf{X}} \in \mathbb{R}^{N\times M} = f_{\text{decoder}}(\hat{W}, \mathbf{Z})$: the reconstructed gene count matrix by the decoder.
$\hat{W}$: parameters of the MAE decoder.
$\phi_k(\mu_k, \Sigma_k, \nu_k)$: the *pdf* of the k-th SMM component.
$\Pi = \{\pi_k, \forall k \in [1, K]\}$: the weights of SMM components.
$\Theta = \{\theta_k : \mu_k, \Sigma_k, \nu_k, \pi_k, \forall k \in [1, K]\}$: parameters of the SMM components.
$\xi_t \in [1, K]$: the SMM component membership of $\mathbf{z}_t$.

We re-write the Student's t distribution of the k-th component of the SMM as a Gaussian scale mixture:

$$\phi((\mathbf{z}_t|\mu_k, \Sigma_k, \nu_k) = \int N(\mathbf{z}_t|\mu_k, \frac{\Sigma_k}{\zeta_{t,k}})\Gamma(\zeta_{t,k}|\frac{\nu_k}{2}, \frac{\nu_k}{2})d\zeta_{t,k}$$

Therefore, under the EM framework, all hidden variables are $H = \{h_i : \xi_i, \zeta_{i,k}, \forall i \in [1, N], \forall k \in [1, K]\}$. The complete data log likelihood is:

$$\mathcal{L}_c(\Theta) = \log P(X, H|\Theta) = \sum_t \sum_k \mathbb{I}(\xi_t = k)(\log \pi_k + \log f(\mathbf{z}_t, \zeta_{t,k}|\mu_k, \Sigma_k, \nu_k))$$

$$\log f(\mathbf{z}_t, \zeta_{t,k}|\mu_k, \Sigma_k, \nu_k) \propto \log \Gamma(\zeta_{t,k}|\frac{\nu_k}{2}, \frac{\nu_k}{2}) + \frac{D}{2}\log \zeta_{t,k} - \frac{1}{2}\log|\Sigma_k| - \frac{1}{2}\zeta_{t,k}\sigma_{t,k}$$

$$\sigma_{t,k} = (\mathbf{z}_t - \mu_k)^T \Sigma_k^{-1}(\mathbf{z}_t - \mu_k)$$

E step. In the t-th iteration, we have the auxiliary function Q as:

$$\begin{aligned} Q(\Theta, \Theta^{(\nu-1)}) &= \mathbb{E}(\mathcal{L}_c(\Theta) \mid \Theta^{(\nu-1)}) \\ &= \sum_t \sum_k p(\xi_t = k \mid \mathbf{z}_t, \Theta^{(\nu-1)})\Big(\log \pi_k^{(\nu-1)} + \mathbb{E}(\log f(z_t, \zeta_{t,k} \mid \mu_k^{(\nu-1)}, \Sigma_k^{(\nu-1)}, \nu_k^{(\nu-1)}))\Big) \end{aligned}$$

The expected sufficient statistics (ESS) are:

$$\bar{\xi}_{t,k}^{(\nu)} = p(\xi_t = k|\mathbf{z}_t, \Theta^{(\nu-1)}) = \frac{\pi_k^{(\nu-1)}\phi(\mathbf{z}_t|\mu_k^{(\nu-1)}, \Sigma_k^{(\nu-1)}, v_k^{(\nu-1)})}{\sum_{k'} \pi_{k'}^{(\nu-1)}\phi(\mathbf{z}_t|\mu_{k'}^{(\nu-1)}, \Sigma_{k'}^{(\nu-1)}, v_{k'}^{(\nu-1)})}$$

$$\bar{\zeta}_{t,k}^{(\nu)} = \mathbb{E}(p(\zeta_{t,k}|\mathbf{z}_t, \theta_k^{(\nu-1)})) = \mathbb{E}\left(\Gamma\left(\zeta_{t,k}\middle|\frac{v_k^{(\nu-1)} + D}{2}, \frac{v_k^{(\nu-1)} + \sigma_{t,k}^{(\nu-1)}}{2}\right)\right) = \frac{v_k^{(\nu-1)} + D}{v_k^{(\nu-1)} + \sigma_{t,k}^{(\nu-1)}}$$

Then the complete data log likelihood of $\mathbf{z}_t$ becomes:

$$\mathbb{E}(\log f(\mathbf{z}_t, \zeta_{t,k}|\mu_k, \Sigma_k, v_k)) \propto G(\mathbf{z}_t, \mu_k, \Sigma_k)^{(\nu)} + F(\zeta_{t,k}, v_k)^{(\nu)}$$

$$G(\mathbf{z}_t, \mu_k, \Sigma_k)^{(\nu)} = -\frac{1}{2}\log|\Sigma_k| - \frac{\bar{\zeta}_{t,k}^{(\nu)}}{2}\sigma_{t,k}$$

$$F(\zeta_{t,k}, v_k)^{(\nu)} = \frac{v_k \log(v_k/2)}{2} - \Gamma(v_k/2) + \frac{v_k}{2}(\log\bar{\zeta}_{t,k}^{(\nu)} - \bar{\zeta}_{t,k}^{(\nu)})$$

M step. In the ν-th iteration, we maximize Q with respect to $\forall\theta_k \in \Theta$. Rather than achieving the MLE, we introduce a prior distribution on θ_k and solve for the MAP of θ_k to alleviate model overfitting. Specifically, we introduce a conjugate prior on Π as a Dirichlet distribution, and a conjugate prior on $\{\mu_k, \Sigma_k\}$ as a normal-inverse Wishart (NIW) distribution:

$$\mathrm{Dir}(\Pi|\alpha^0) \equiv \frac{1}{B(\alpha^0)}\prod_k \pi_k^{\alpha_k^0-1},$$

$$\begin{aligned}\mathrm{NIW}(\mu_k, \Sigma_k \mid m_0, \kappa_0, S_0, \rho_0) \propto |\Sigma_k|^{-1/2}\exp\left(-\frac{\kappa_0}{2}(\mu_k - m_0)^T\Sigma_k^{-1}(\mu_k - m_0)\right) \\ \times |\Sigma_k|^{-(\rho_0+D+1)/2}\exp\left(-\frac{1}{2}\mathrm{tr}(S_0\Sigma_k^{-1})\right)\end{aligned}$$

Here, we have weaker priors as $\alpha^0 = \vec{1}$, $\rho_0 = D{+}2$, $S_0 = N^{-1}\mathrm{diag}\left((Z - \frac{1}{N}\Omega Z)^T(Z - \frac{1}{N}\Omega Z)\right)$, $\Omega = \vec{1}^T \times \vec{1}$, $\kappa_0 = 0$, and $m_0 = \frac{\sum_t \mathbf{z}_t}{N}$. The posterior distribution of Π and $\{\mu_k, \Sigma_k\}$ are:

$$p(\Pi|X) \sim \mathrm{Dir}(\Pi|\alpha^{(\nu)}) \equiv \frac{1}{B(\alpha^{(\nu)})}\prod_k \pi_k^{\alpha_k^{(\nu)}-1}$$

$$\alpha_k^{(\nu)} = \alpha_k^0 + \sum_t \bar{\xi}_{t,k}^{(\nu)}, \forall k \in [1, K]$$

$$p(\{\mu_k, \Sigma_k\}|X) \sim \mathrm{NIW}(\mu_k, \Sigma_k|m_k^{(\nu)}, \kappa_k^{(\nu)}, S_k^{(\nu)}, \rho_k^{(\nu)})$$

$$\kappa_k^{(\nu)} = \kappa_0 + \bar{\omega_k}^{(\nu)} = \bar{\omega_k}^{(\nu)}$$

$$\bar{\omega}_{t,k}^{(\nu)} = \bar{\xi}_{t,k}^{(\nu)} \bar{\zeta}_{t,k}^{(\nu)}$$

$$\bar{\omega_k}^{(\nu)} = \sum_t \bar{\omega}_{t,k}^{(\nu)}$$

$$\rho_k^{(\nu)} = \rho_0 + \bar{\xi_k}^{(\nu)}$$

$$\bar{\xi_k}^{(\nu)} = \sum_t \bar{\xi}_{t,k}^{(\nu)}$$

$$m_k^{(\nu)} = \frac{\bar{\omega_k}^{(\nu)} \bar{z_k}^{(\nu)} + \kappa_0 m_0}{\kappa_k^{(\nu)}} = \bar{\mathbf{z}}_k^{(\nu)}$$

$$\bar{\mathbf{z}}_k^{(\nu)} = \frac{\sum_t (\bar{\omega}_{t,k}^{(\nu)} \mathbf{z}_t)}{\bar{\omega_k}^{(\nu)}}$$

$$S_k^{(\nu)} = S_0 + \sum_t \left(\bar{\omega}_{t,k}^{(\nu)} \mathbf{z}_t \mathbf{z}_t^T + \kappa_0 m_0 m_0^T - \kappa_k^{(\nu)} m_k^{(\nu)} (m_k^{(\nu)})^T \right) = S_0 + \sum_t \left(\bar{\omega}_{t,k}^{(\nu)} \mathbf{z}_t \mathbf{z}_t^T - \kappa_k^{(\nu)} m_k^{(\nu)} (m_k^{(\nu)})^T \right)$$

Then we have the MAP estimates of π_k and $\{\mu_k, \Sigma_k\}$ as $\pi_k^{(\nu)}$ and $\{\mu_k^{(\nu)}, \Sigma_k^{(\nu)}\}$:

$$\pi_k^{(\nu)} = \frac{\alpha_k^{(\nu)} - 1}{\sum_{k'} \alpha_{k'}^{(\nu)} - K}$$

$$\mu_k^{(\nu)} = m_k^{(\nu)} = \bar{\mathbf{z}}_k^{(\nu)}$$

$$S_k^{(\nu)} = S_0 + \sum_t \left[\bar{\omega}_{t,k}^{(\nu)} (\mathbf{z}_t - m_k^{(\nu)})(\mathbf{z}_t - m_k^{(\nu)})^T \right] = S_0 + S_{\text{mle},k}^{(\nu)}$$

$$S_{\text{mle},k}^{(\nu)} = \sum_t \left[\bar{\omega}_{t,k}^{(\nu)} (\mathbf{z}_t - \bar{\mathbf{z}}_k^{(\nu)})(\mathbf{z}_t - \bar{\mathbf{z}}_k^{(\nu)})^T \right]$$

$$\Sigma_k^{(\nu)} = \frac{S_k^{(\nu)}}{\rho_k^{(\nu)} + D + 2} = \frac{S_0 + S_{\text{mle},k}^{(\nu)}}{\hat{\rho_0} + \bar{\xi_k}^{(\nu)}} = \frac{\hat{\rho_0}}{\hat{\rho_0} + \bar{\xi_k}^{(\nu)}} \cdot \frac{S_0}{\hat{\rho_0}} + \frac{\bar{\xi_k}^{(\nu)}}{\hat{\rho_0} + \bar{\xi_k}^{(\nu)}} \cdot \frac{S_{\text{mle},k}^{(\nu)}}{\bar{\xi}_k^{(\nu)}}$$

$$\Sigma_k^{(\nu)}(t,j) = \begin{cases} \hat{\beta}\Sigma_0(t,j) + (1-\hat{\beta})\Sigma_{\text{mle},k}^{(\nu)}(t,j), & \text{if } t = j \\ (1-\hat{\beta})\Sigma_{\text{mle},k}^{(\nu)}(t,j), & \text{otherwise} \end{cases}$$

The off-diagonal entries in $\Sigma_k^{(\nu)}$ are shrunk toward 0 to promote its sparsity, thereby reducing the computational load and possibility of overfitting.

$v_k^{(\nu)}$ can be derived by maximizing $\sum_t(\bar{\xi}_{t,k}^{(\nu)} \cdot F(\zeta_{t,k}^{(\nu)}, v_k^{(\nu)}))$. However, there is no closed-form solution, so we apply the generalized EM (GEM) to approximate the solution as follows:

$$\zeta_{t,k}^{(\nu)} \sim \Gamma\left(\zeta_{t,k}^{(\nu)} \middle| \frac{v_k^{(\nu-1)} + D}{2}, \frac{v_k^{(\nu-1)} + \sigma_{t,k}^{(\nu-1)}}{2}\right)$$

$$\Rightarrow \log\bar{\zeta}_{t,k}^{(\nu)} = \mathbb{E}(\log \zeta_{t,k}^{(\nu)}) = \Psi\left(\frac{v_k^{(\nu-1)} + D}{2}\right) - \log\left(\frac{v_k^{(\nu-1)} + \sigma_{t,k}^{(\nu-1)}}{2}\right)$$

where $\Psi(x) \equiv \frac{d}{dx} \log \Gamma(x)$ is the digamma function. Then we have:

$$\frac{d}{dv_k^{(\nu)}} \sum_t \left(\bar{\xi}_{t,k}^{(\nu)} \cdot F(\zeta_{t,k}^{(\nu)}, v_k^{(\nu)})\right) = \sum_t \left[\bar{\xi}_{t,k}^{(\nu)} \left(\frac{1}{2} \log\left(\frac{v_k^{(\nu)}}{2}\right) + \frac{1}{2} - \frac{1}{2}\Psi\left(\frac{v_k^{(\nu)}}{2}\right) + \frac{1}{2}(\log \bar{\zeta}_{t,k}^{(\nu)} - \bar{\zeta}_{t,k}^{(\nu)})\right)\right]$$

Then $v_k^{(\nu)} \leftarrow v_k^{(\nu)} - \lambda \cdot \frac{d}{dv_k^{(\nu)}} F(\zeta_{t,k}^{(\nu)}, v_k^{(\nu)})$ is repeated for several times to achieve a "partial" improvement to $v_k^{(\nu)}$, which still guarantees convergence to a local optimum. Next, the EM algorithm continues to the E step of the $(\nu + 1)$-th iteration to update $H^{(\nu+1)} = \{h_{t,k}^{(\nu+1)} : \bar{\xi}_{t,k}^{(\nu+1)}, \bar{\zeta}_{t,k}^{(\nu+1)}, \forall t \in [1, N], \forall k \in [1, K]\}$ until either convergence is achieved, or a pre-specified number of iterations is reached. Finally, the score and soft assignment of $\mathbf{z}_t$ to the k-th component $(q_{t,k})$ can be calculated by plugging in θ_k as:

$$q_{t,k} = \pi_k \phi(\mathbf{z}_t | \mu_k, \Sigma_k, v_k)$$

$$q_{t,k} = \frac{q_{t,k}}{\sum_j q_{t,j}}, \forall t \in [1, N], \forall k \in [1, K]$$

Note 7 Deriving the Seeding Gene-Gene Similarity Matrix

During the training of the model, an initial gene-gene similarity matrix is incorporated into the loss function $\mathcal{L}_1$ and $\mathcal{L}_2$ as a regularization term to inform the initial training phase of the model. We leverage multiple image recognition operators to extract feature descriptors from images of gene spatial expression maps, based on which the seeding gene-gene similarity matrix is calculated. Specifically, on the gray-scale level of the image, we utilize Sobel operator to extract gradient magnitude and orientation descriptors, Laplacian operator to extract gradient divergence descriptor, and Canny operators to extract the gradient continuity descriptor. Meanwhile, three average and standard deviation pooling filters of different sizes are used to extract patch brightness descriptors. The normalized spatial expression matrix of gene u is denoted as $X^u \in \mathbb{R}^{N_x \times N_y}$, where N_x and N_y denote the number of spatial spots along the horizontal and vertical directions of the spatial map. Out-of-tissue spatial spots are all padded with 0s. X^u is smoothened with a convolutional Gaussian kernel $H \in \mathbb{R}^{d \times d}$, obtaining a denoised expression matrix $\tilde{X}^u$:

$$H_{i,j} = \frac{1}{2\pi\sigma^2} \exp\left(-\frac{(i-(k-1)/2)^2 + (j-(k-1)/2)^2}{2\sigma^2}\right), \quad 1 \le i, j \le d;$$

$$\tilde{X}^u_{i,j} = \sum (H \odot X^u_{s(k),t(k)});$$

Next, Sobel, Laplacian, and Canny operators are applied on $\tilde{X}^u$ to generate matrices of corresponding descriptors. For a specific gene u, let G^u and Θ^u denote the matrices of gradient magnitude and orientation descriptors, L^u the matrix of gradient divergence descriptor, and C^u the matrix of gradient continuity descriptor. The pooling filters are applied by segmenting $\tilde{X}^u$ into small patches containing $k \times k$ spots, where $k \in \{1, 3, 5\}$, from which three patch brightness mean matrices $A^u(k)$ and two variance matrices $S^u(k)$ (eligible only for $k = 3, 5$) are calculated:

$$A^u_{i,j}(k) = \text{avg}(X^u_{s(k),t(k)}), \quad S^u_{i,j}(k) = \text{std}(X^u_{s(k),t(k)}), \quad 1 \le i \le N_x, 1 \le j \le N_y$$

$$s(k) = \left[i - \frac{k-1}{2} : i + \frac{k-1}{2}\right], \quad t(k) = \left[j - \frac{k-1}{2} : j + \frac{k-1}{2}\right]$$

Finally, the initial gene similarity matrix S is calculated as the average Pearson correlation between gene pairs' descriptor matrices:

$$S_{u,v} = \begin{cases} \text{avg}(\rho_{u,v}(\Xi^u, \Xi^v)); \Xi \in \{A(k), S(k), G, \Theta, L, C\}, k \in 1, 3, 5 & \text{if } u \ne v \\ 0 & \text{if } u = v \end{cases}$$

Here, u, v represent any two genes in the dataset.

Note 8 The Joint Optimization of the Gene Representation Learning and SMM via a Discriminative Boosted Clustering

The two loss functions, $\mathcal{L}_1$ and $\mathcal{L}_2$, are designed to optimize different components: $\mathcal{L}_1$ focuses on optimizing the MAE, while $\mathcal{L}_2$ optimizes both the MAE and the SMM together. This iterative process progressively improves the clustering-oriented gene embeddings and clustering results. Upon completing the inference of SMM parameters $\widetilde{\Theta}$ in each epoch, an epoch-level loss $\mathcal{L}_1$ is calculated for updating parameters of MAE:

$$\mathcal{L}_1 = -\mathcal{L}_{\ell\ell} + \eta_1 \mathcal{L}_{\ell ap} - \eta_2 \mathcal{L}_{\text{size}} + \eta_3 \mathcal{L}_r. \tag{16}$$

Here, $\mathcal{L}_{\ell ap}$ is a Laplacian regularization term that promotes the similarities among gene embeddings $\mathbf{Z}$ to be consistent with a seeding gene-gene similarity matrix $\mathcal{S}$, informing the initial training phase. The derivation of $\mathcal{S}$ is detailed in Note 7. $\mathcal{L}_{\ell ap}$ is defined as follows:

$$\mathcal{L}_{\ell ap} = Tr\left(Z^T\left(I - \mathcal{D}^{-\frac{1}{2}}\mathcal{S}\mathcal{D}^{-\frac{1}{2}}\right)Z\right), \tag{17}$$

where $\mathcal{D}$ is the degree matrix of $\mathcal{S}$. $\mathcal{L}_{\ell\ell}$ represents the log likelihood of the embeddings given the estimated SMM parameters $\widetilde{\Theta}$:

$$\mathcal{L}_{\ell\ell} = \sum_{\text{t=1}}^{\text{N}} \log\left[\sum_k q_{t,k}\right], \tag{18}$$

$$q_{t,k} = \pi_k \phi(\mathbf{z}_t | \mu_k, \Sigma_k, \nu_k), \forall t \in [1, N], \forall k \in [1, K]. \tag{19}$$

$\mathcal{L}_{size}$ penalizes empty and tiny clusters, while exempting those whose size exceeds a predefined threshold υ so that image assignments is not overly uniform:

$$\mathcal{L}_{size} = \sum_{k=1}^{K} -J_k \log J_k, J_k = \begin{cases} \frac{\Sigma_t^N q_{t,k}}{N} & , if J_k \le \upsilon \\ 1 & , otherwise \end{cases} \tag{20}$$

$\mathcal{L}_r$, defined in Equation (2) of main paper, aims to enhance the local-context awareness of embeddings. We set $\eta_1 = 0.5$, $\eta_2 = 0.1$, $\eta_3 = 0.1$. Note, the value of η_1 decays as the training progresses so that the impact of the seeding matrix diminishes over the training course. Subsequently, within the same epoch, a batch-level loss $\mathcal{L}_2 = \mathcal{L}_c + \lambda_1 \mathcal{L}_r + \lambda_2 \mathcal{L}_{\ell ap}$ is utilized to update MAE and SMM parameters across successive batches. Here, $\mathcal{L}_r$ remains same as in Equation (2) of main paper, except being calculated on the batch-level. $\mathcal{L}_c$ boosts high-confidence images, incrementally grouping similar instances while separating dissimilar ones:

$$\mathcal{L}_c = KL(\mathcal{P}|\mathcal{Q}) = \sum_t^N \sum_j^K \mathfrak{p}_{t,j} \log \frac{\mathfrak{p}_{t,j}}{\mathfrak{q}_{t,j}}, \tag{21}$$

$$\text{where } \mathfrak{q}_{t,k} = \frac{q_{t,k}}{\sum_c q_{t,c}}, \mathfrak{p}_{t,k} = \frac{\mathfrak{q}_{t,k}^2 / \sum_t \mathfrak{q}_{t,k}}{\sum_c \left(\mathfrak{q}_{t,c}^2 / \sum_t \mathfrak{q}_{t,c} \right)} \tag{22}$$

Here, $q_{t,k}$ is same as in Equation 19, $\mathfrak{q}_{t,k}$ represents the probability of assigning t-th image to the k-th SMM component, and $\mathfrak{p}_{t,k}$ an auxiliary target distribution that boosts up high-confidence images. After this joint optimization, the training progresses to the next epoch, iterating until the end of the training process.

Then, we focus on deriving the gradients of $\mathcal{L}_{\ell\ell}$ and $\mathcal{L}_{size}$ with respect to $\mathbf{Z}$, and the gradients of $\mathcal{L}_c$ with respect to $\mathbf{Z}$ and $\mathbf{\Theta}$. The derivations of the gradients of L_{lapl} and L_r with respect to $\mathbf{Z}$ and the gradient of L_r with respect to $\hat{\mathbf{X}}$ are relatively trivial and therefore ignored.

$$\mathcal{L}_{\ell\ell} = \log P(\mathbf{Z}|\mathbf{\Theta}) = \sum_{t=1}^{N} \log \left[\sum_k q_{t,k} \right]$$

$$\mathcal{L}_c = KL(P||Q) = \sum_{t=1}^{N} \sum_{j=1}^{K} p_{t,j} \log \frac{p_{t,j}}{q_{t,j}}$$

$$p_{t,k} = \frac{q_{t,k}^2 / \sum_t q_{t,j}}{\sum_j \left(q_{t,j}^2 / \sum_t q_{t,j} \right)}$$

$$\mathcal{L}_{size}(\mathbf{Z}, \mathbf{\Theta}) = \sum_{k=1}^{K} -J_k \log J_k$$

where

$$J_k = \begin{cases} \frac{\sum_{t=1}^{N} q_{t,k}}{N}, & \text{if } J_k \le \tau \\ 1, & \text{otherwise} \end{cases}$$

The density function of $\mathbf{z}_t$ given $\{\mu_k, \Sigma_k, \nu_k\}$ is:

$$\phi(\mathbf{z}_t|\mu_k, \Sigma_k, \nu_k) \propto \frac{\Gamma\left(\frac{\nu_k+D}{2}\right)}{\Gamma\left(\frac{\nu_k}{2}\right)} \nu_k^{-D/2} |\Sigma_k|^{-1/2} \left[1 + \frac{1}{\nu_k} (\mathbf{z}_t - \mu_k)^T \Sigma_k^{-1} (\mathbf{z}_t - \mu_k) \right]^{-\frac{\nu_k+D}{2}}$$

$$= h(\nu_k) |\Sigma_k|^{-1/2} \left[1 + \frac{\sigma_{t,k}}{\nu_k} \right]^{-\frac{\nu_k+D}{2}} = h(\nu_k) |\Sigma_k|^{-1/2} u_{t,k}^{-\frac{\nu_k+D}{2}}$$

$$\frac{\partial u_{t,k}}{\partial \mathbf{z}_t} = \frac{2}{\nu_k} \Sigma_k^{-1} (\mathbf{z}_t - \mu_k)$$

$$\Rightarrow \frac{\partial q_{t,k}}{\partial \mathbf{z}_t} = \frac{\partial q_{t,k}}{\partial u_{t,k}} \cdot \frac{\partial u_{t,k}}{\partial \mathbf{z}_t} = -\pi_k h(\nu_k) |\Sigma_k|^{-1/2} \left(\frac{\nu_k + D}{2} \right) u_{t,k}^{-\left(\frac{\nu_k+D}{2}+1\right)} \cdot \frac{2}{\nu_k} \Sigma_k^{-1} (\mathbf{z}_t - \mu_k)$$

$$= -\frac{v_k + D}{v_k} u_{t,k}^{-1} q_{t,k} \cdot \Sigma_k^{-1} (\mathbf{z}_t - \mu_k)$$

$$\frac{\partial q_{t,j}}{\partial \mu_k} = \begin{cases} \frac{\partial q_{t,k}}{\partial u_{t,k}} \cdot \frac{\partial u_{t,k}}{\partial \mu_k} = -\frac{(v_k+D)}{v_k} u_{t,k}^{-1} q_{t,k} \cdot \Sigma_k^{-1} (\mu_k - \mathbf{z}_t), & j = k \\ 0, & j \neq k \end{cases}$$

$$\frac{\partial q_{t,j}}{\partial \Sigma_k} = \begin{cases} \frac{\partial q_{t,k}}{\partial u_{t,k}} \cdot \frac{\partial u_{t,k}}{\partial \Sigma_k} + \frac{\partial q_{t,k}}{\partial |\Sigma_k|} \cdot \frac{\partial |\Sigma_k|}{\partial \Sigma_k}, & j = k \\ 0, & j \neq k \end{cases}$$

$$= \begin{cases} q_{t,k} \left(\frac{(v_k+D)}{2v_k} u_{t,k}^{-1} \cdot \Sigma_k^{-1} (\mathbf{z}_t - \mu_k)(\mathbf{z}_t - \mu_k)^T \Sigma_k^{-1} - \frac{1}{2} \Sigma_k^{-1} \right) = q_{t,k} f(\mathbf{z}_t, \mu_k, v_k, \Sigma_k, D), & j = k \\ 0, & j \neq k \end{cases}$$

$$\frac{\partial q_{t,j}}{\partial v_k} = \begin{cases} q_{t,k} \frac{\partial \ln(q_{t,k})}{\partial v_k}, & j = k \\ 0, & j \neq k \end{cases}$$

$$= \begin{cases} q_{t,k} \left(\frac{v_k+D}{2} u_{t,k}^{-1} \frac{\sigma_{t,k}}{v_k^2} - \frac{1}{2} \ln u_{t,k} + \frac{1}{2} \Gamma \left(\frac{v_k+D}{2} \right) \Psi \left(\frac{v_k+D}{2} \right) - \frac{1}{2} \Gamma \left(\frac{v_k}{2} \right) \Psi \left(\frac{v_k}{2} \right) - \frac{D}{2v_k} \right), & j = k \\ 0, & j \neq k \end{cases}$$

$$= \begin{cases} q_{t,k} \mathcal{G}(\mathbf{z}_t, \mu_k, v_k, \Sigma_k, D), & j = k \\ 0, & j \neq k \end{cases}$$

$$\frac{\partial q_{t,j}}{\partial \pi_k} = \begin{cases} \frac{q_{t,k}}{\pi_k}, & j = k \\ 0, & j \neq k \end{cases}$$

By the chain rule of derivatives, we have:

$$\frac{\partial \mathfrak{q}_{t,k}}{\partial \mathbf{z}_t} = \frac{\left(\frac{\partial q_{t,k}}{\partial \mathbf{z}_t} \cdot \sum_j q_{t,j} - q_{t,k} \cdot \sum_j \frac{\partial q_{t,j}}{\partial \mathbf{z}_t} \right)}{\left(\sum_j q_{t,j} \right)^2}$$

$$= -\frac{\nu_k + D}{\nu_k} \mathbb{q}_{t,k} \left(u_{t,k}^{-1} \cdot \Sigma_k^{-1} (\mathbf{z}_t - \mu_k) - \sum_j u_{t,j}^{-1} \mathbb{q}_{t,j} \cdot \Sigma_j^{-1} (\mathbf{z}_t - \mu_j) \right)$$

$$\frac{\partial \mathbb{q}_{t,j}}{\partial \mu_k} = \begin{cases} \frac{\left(\frac{\partial q_{t,j}}{\partial \mu_k} \cdot \sum_j q_{t,j} - q_{t,j} \cdot \sum_j \frac{\partial q_{t,j}}{\partial \mu_k} \right)}{\left(\sum_j q_{t,j} \right)^2}, & j \neq k \\ \frac{\left(\frac{\partial q_{t,k}}{\partial \mu_k} \cdot \sum_j q_{t,j} - q_{t,k} \cdot \sum_j \frac{\partial q_{t,j}}{\partial \mu_k} \right)}{\left(\sum_j q_{t,j} \right)^2}, & j = k \end{cases}$$

$$= \begin{cases} \frac{\nu_k + D}{\nu_k} \mathbb{q}_{t,j} \left(u_{t,k}^{-1} \mathbb{q}_{t,k} \cdot \Sigma_k^{-1} (\mu_k - \mathbf{z}_t) \right), & j \neq k \\ -\frac{\nu_k + D}{\nu_k} \mathbb{q}_{t,k} \left(u_{t,k}^{-1} \cdot \Sigma_k^{-1} (\mu_k - \mathbf{z}_t) - u_{t,k}^{-1} \mathbb{q}_{t,k} \cdot \Sigma_k^{-1} (\mu_k - \mathbf{z}_t) \right), & j = k \end{cases}$$

$$\frac{\partial \mathbb{q}_{t,j}}{\partial \Sigma_k} = \begin{cases} \frac{\left(\frac{\partial q_{t,j}}{\partial \Sigma_k} \cdot \sum_j q_{t,j} - q_{t,j} \cdot \sum_j \frac{\partial q_{t,j}}{\partial \Sigma_k} \right)}{\left(\sum_j q_{t,j} \right)^2}, & j \neq k \\ \frac{\left(\frac{\partial q_{t,k}}{\partial \Sigma_k} \cdot \sum_j q_{t,j} - q_{t,k} \cdot \sum_j \frac{\partial q_{t,j}}{\partial \Sigma_k} \right)}{\left(\sum_j q_{t,j} \right)^2}, & j = k \end{cases}$$

$$= \begin{cases} -\mathbb{q}_{t,j} \mathbb{q}_{t,k} f(\mathbf{z}_t, \mu_k, \nu_k, \Sigma_k, D), & j \neq k \\ \mathbb{q}_{t,k} \left(f(\mathbf{z}_t, \mu_k, \nu_k, \Sigma_k, D) - \mathbb{q}_{t,k} \cdot f(\mathbf{z}_t, \mu_k, \nu_k, \Sigma_k, D) \right), & j = k \end{cases}$$

$$\frac{\partial \mathbb{q}_{t,j}}{\partial \nu_k} = \begin{cases} \frac{\left(\frac{\partial q_{t,j}}{\partial \nu_k} \cdot \sum_j q_{t,j} - q_{t,j} \cdot \sum_j \frac{\partial q_{t,j}}{\partial \nu_k} \right)}{\left(\sum_j q_{t,j} \right)^2} = -\mathbb{q}_{t,j} \mathbb{q}_{t,k} g(\mathbf{z}_t, \mu_k, \nu_k, \Sigma_k, D), & j \neq k \\ \frac{\left(\frac{\partial q_{t,k}}{\partial \nu_k} \cdot \sum_j q_{t,j} - q_{t,k} \cdot \sum_j \frac{\partial q_{t,j}}{\partial \nu_k} \right)}{\left(\sum_j q_{t,j} \right)^2} = \mathbb{q}_{t,k} \left(1 - \mathbb{q}_{t,k} \right) g(\mathbf{z}_t, \mu_k, \nu_k, \Sigma_k, D), & j = k \end{cases}$$

$$\frac{\partial \mathbb{q}_{t,j}}{\partial \pi_k} = \begin{cases} \frac{\left(\frac{\partial q_{t,j}}{\partial \pi_k} \cdot \sum_j q_{t,j} - q_{t,j} \cdot \sum_j \frac{\partial q_{t,j}}{\partial \pi_k} \right)}{\left(\sum_j q_{t,j} \right)^2} = -\frac{\mathbb{q}_{t,j} \mathbb{q}_{t,k}}{\pi_k}, & j \neq k \\ \frac{\left(\frac{\partial q_{t,k}}{\partial \pi_k} \cdot \sum_j q_{t,j} - q_{t,k} \cdot \sum_j \frac{\partial q_{t,j}}{\partial \pi_k} \right)}{\left(\sum_j q_{t,j} \right)^2} = \frac{\mathbb{q}_{t,k} \left(1 - \mathbb{q}_{t,k} \right)}{\pi_k}, & j = k \end{cases}$$

Then the derivatives of $\mathcal{L}_{\ell\ell}$ and $\mathcal{L}_{size}$ with respect to $\mathbf{z}_t$ are:

$$\frac{\partial \mathcal{L}_{\ell\ell}}{\partial \mathbf{z}_t} = \frac{\sum_j \frac{\partial q_{t,j}}{\partial \mathbf{z}_t}}{\sum_j q_{t,j}} = -\frac{\nu_k + D}{\nu_k} \frac{u_{t,k}^{-1} q_{t,k} \cdot \Sigma_k^{-1} (\mathbf{z}_t - \mu_k)}{\sum_j q_{t,j}}$$

$$\frac{\partial \mathcal{L}_{size}}{\partial \mathbf{z}_t} = \sum_{j \in \{J_j \le \tau\}} \left[-(1 + \log J_j) \frac{1}{N} \sum_{i=1}^{N} \frac{\partial \mathbb{q}_{t,j}}{\partial \mathbf{z}_t} \right]$$

As the target distribution P is fixed during the joint optimization within an epoch, we have the derivatives of $\mathcal{L}_c$ with respect to $\mathbf{z}_t$ and $\theta_k = \{\mu_k, \Sigma_k, v_k, \pi_k\}$ as:

$$\frac{\partial \mathcal{L}_c}{\partial \mathbf{z}_t} = -\sum_j \frac{\mathbb{p}_{t,j}}{\mathbb{q}_{t,j}} \cdot \frac{\partial \mathbb{q}_{t,j}}{\partial \mathbf{z}_t} = \frac{v_k + D}{v_k} \sum_j (\mathbb{p}_{t,k} - \mathbb{q}_{t,k}) u_{t,k}^{-1} \cdot \Sigma_k^{-1} (\mathbf{z}_t - \mu_k)$$

$$\frac{\partial \mathcal{L}_c}{\partial \mu_k} = -\sum_t \sum_j \frac{\mathbb{p}_{t,j}}{\mathbb{q}_{t,j}} \cdot \frac{\partial \mathbb{q}_{t,j}}{\partial \mu_k} = \frac{v_k + D}{v_k} \sum_t (\mathbb{p}_{t,k} - \mathbb{q}_{t,k}) u_{t,k}^{-1} \cdot \Sigma_k^{-1} (\mu_k - \mathbf{z}_t)$$

$$\frac{\partial \mathcal{L}_c}{\partial \Sigma_k} = -\sum_t \sum_j \frac{\mathbb{p}_{t,j}}{\mathbb{q}_{t,j}} \cdot \frac{\partial \mathbb{q}_{t,j}}{\partial \Sigma_k} = \sum_t (\mathbb{q}_{t,k} - \mathbb{p}_{t,k}) f(\mathbf{z}_t, \mu_k, v_k, \Sigma_k, D)$$

$$\frac{\partial \mathcal{L}_c}{\partial v_k} = -\sum_t \sum_j \frac{\mathbb{p}_{t,j}}{\mathbb{q}_{t,j}} \cdot \frac{\partial \mathbb{q}_{t,j}}{\partial v_k} = -\sum_t (\mathbb{p}_{t,k} - \mathbb{q}_{t,k}) g(\mathbf{z}_t, \mu_k, v_k, \Sigma_k, D)$$

$$\frac{\partial \mathcal{L}_c}{\partial \pi_k} = -\sum_t \sum_j \frac{\mathbb{p}_{t,j}}{\mathbb{q}_{t,j}} \cdot \frac{\partial \mathbb{q}_{t,j}}{\partial \pi_k} = -\sum_t \frac{\mathbb{p}_{t,k} - \mathbb{q}_{t,k}}{\pi_k}$$

Note 9 SIGEL-SPS

Based on the similarity between SRGs of real gene and simulated genes with designated expression patterns, spatially patterned genes can be accurately pinpointed. We model the spatial expression of a given gene using a NB distribution, with parameters estimated from the designated pattern using a Generalized Linear Model (GLM)-based method. The estimated parameters are then used to simulate genes with desired expression patterns. Specifically, let G represents the set of all genes and S the set of all spots in the dataset. The normalized read counts of gene $t \in G$ at spot $j \in S$, denoted as $X_{t,j}$, follow a NB distribution: $X_{t,j} \sim \text{NB}(\mu_t, s_t)$, where μ_t and s_t represent the mean and dispersion. The variance σ_t^2 and squared coefficient of variation (SCV) for gene t are given by $\sigma_t^2 = \mu_t + \frac{\mu_t^2}{s_t}$ and $\text{cv}_t^2 = \frac{1}{\mu_t} + \frac{1}{s_t}$. We have:

$$\mathbb{E}[\hat{\text{cv}}_t^2] \approx a_0 + \frac{a_1}{\hat{\mu}_t},\ \hat{\mu}_t = \frac{\sum_j X_{t,j}}{N},\ \hat{\text{cv}}_t^2 = \frac{\sum_j (X_{t,j} - \hat{\mu}_t)^2}{(N-1)\hat{\mu}_t^2}, \tag{23}$$

where $\hat{\text{cv}}_t^2$ and $\hat{\mu}_t$ are sample SCV and mean, respectively. N denotes the total number of spots in the ST dataset. Note that $\hat{\text{cv}}_t^2$ approximately follows a χ^2 or gamma distribution. By taking $\hat{\text{cv}}_t^2$ as observations, $\frac{1}{\hat{\mu}_t}$ as inputs, a_0 and a_1 are coefficients, we can use a GLM from the gamma family with an identity link function to perform the regression:

$$\begin{aligned}&\sum_t \log\left(P\left(\hat{\text{cv}}_t^2 \mid \alpha, \beta_t, a_0, a_1\right)\right)\\ &\quad = \sum_i \left(-\alpha \log(\beta_t) - \frac{\hat{\text{cv}}_t^2}{\beta_t} + (\alpha - 1)\log(\hat{\text{cv}}_t^2)\right) + C_1,\end{aligned} \tag{24}$$

$$\mathbb{E}[\hat{\text{cv}}_t^2 \mid \alpha, \beta_t] = \alpha\beta_t = \eta_t = a_0 + \frac{a_1}{\hat{\mu}_t}, \tag{25}$$

where $\alpha = \frac{1}{\sigma}$ is a constant, and σ denotes the GLM dispersion parameter. Let the natural parameter of the GLM be $\theta_t = -1/\mathbb{E}[\hat{\text{cv}}_t^2 \mid \alpha, \beta_t]$, then we have:

$$\begin{aligned}&\sum_i \log\left(P\left(\hat{\text{cv}}_t^2 \mid \alpha, \beta_t, a_0, a_1\right)\right)\\ &\quad \propto \sum_i \left(\frac{\theta_t \hat{\text{cv}}_t^2 - \log\left(-\frac{1}{\theta_i}\right)}{\sigma} + \frac{1-\sigma}{\sigma}\log(\hat{\text{cv}}_t^2)\right),\end{aligned} \tag{26}$$

from which coefficients a_0 and a_1 can be estimated using the Fisher-scoring method (Note 9.1). Assume we aim to simulate a gene with top $t\%$ expression in region $S_1 \subset S$ and NB distribution parameters μ and s. We first calculate and rank the average expressions of all genes in the target dataset, denoted L. The mean μ and cv can be estimated as

$\mu = L@t\%$, and $\mathrm{cv}^2 \approx a_0 + \frac{a_1}{\mu}$. The dispersion s is computed as:

$$s = \frac{1}{(\mathrm{cv}^2 - \frac{1}{\mu})} \tag{27}$$

Let N_1 denote the number of spots in S_1. We simulate N_1 observations from $\mathrm{NB}(\mu, s)$, reorder them based on their simulated read counts. The spots in S_1 are also ranked based on their average expression levels across all genes. The simulated observations are then assigned to spots of the same rank to generate the simulated gene, thus ensuring the original spatial structure is preserved. This process can be repeated to generate simulated genes at specified quantile levels in any tissue region. Finally, the gene most similar to the simulated gene in SGRs is identified as a spatially patterned gene.

Note 9.1 Fisher Scoring Method for Estimating GLM Parameters

By taking $y_t := \hat{\mathrm{cv}}_t^2$ as observations, $x_t := [1, \frac{1}{\hat{\mu}_t}]'$ as inputs, and $w := [a_0, a_1]$ as coefficients, we use a GLM from the gamma family with parameters α and β_t and an identity link function to perform the regression. Then, we have:

$$\mu_{y_t} := \mathbb{E}[\hat{\mathrm{cv}}_t^2 \mid \alpha, \beta_t] = \alpha\beta_t = \eta_t = a_0 + \frac{a_1}{\hat{\mu}_t} = x_t'w. \tag{28}$$

Let $\sigma = \frac{1}{\alpha}$ and θ_t denote the dispersion parameter and the natural parameter of the GLM, respectively. θ_t is related to μ_{y_t} through:

$$\theta_t = -\frac{1}{\mathbb{E}[\hat{\mathrm{cv}}_t^2 \mid \alpha, \beta_t]} = -\frac{1}{\mu_{y_t}} = -\frac{1}{\alpha\beta_t}. \tag{29}$$

Then, log-likelihood function of the GLM can be written as:

$$\ell(y|w) = \sum_t \log\left(P\left(y_t \mid \alpha, \beta_t, w\right)\right) \propto \sum_t \left(\frac{\theta_t y_t - A(\theta_t)}{\sigma} + \frac{1-\sigma}{\sigma}\log(y_t)\right), \tag{30}$$

where $A(\theta_t) = \log\left(-\frac{1}{\theta_t}\right)$. Then, we have:

$$\mu_{y_t} = \frac{\partial A(\theta_t)}{\partial \theta_t}, \quad Var(y_t) = \frac{\partial^2 A(\theta_t)}{\partial \theta_t^2}\sigma = \frac{\partial \mu_{y_t}}{\partial \theta_t}\sigma. \tag{31}$$

We aim to maximize the log-likelihood function with respect to the parameters w:

$$\max_w \ell(y|w). \tag{32}$$

We utilizes the Newton-Raphson algorithm to find the optimal w with the gradient

vector $g(w)$ and the Hessian matrix $H(w)$. $g(w)$ is computed as:

$$g(w) = \frac{\partial \ell(y|w)}{\partial w} = \frac{1}{\sigma}\sum_{t=1}^{N}(y_t - \mu_{y_t})\frac{\partial \theta_t}{\partial \mu_{y_t}}\frac{\partial \mu_{y_t}}{\partial \eta_t}x_t, \tag{33}$$

where $\frac{\partial \theta_t}{\partial \mu_{y_t}} = \frac{1}{\mu_{y_t}^2} = \theta_t^2$ and $\frac{\partial \mu_{y_t}}{\partial \eta_t} = 1$. Then $g(w)$ simplifies to:

$$g(w) = \frac{1}{\sigma}X\left((y - \mu_y) \odot \theta^2\right), \tag{34}$$

where $X = [x_1, \ldots, x_N], y = [y_1, .., y_N]', \mu_y = [\mu_{y_1}..\mu_{y_N}]'$, and $\theta = [\theta_1..\theta_t]'$. $\odot$ denotes element-wise multiplication. $H(w)$ is computed as:

$$H(w) = \frac{\partial^2 \ell(y|w)}{\partial w^2}. \tag{35}$$

w is updated in one iteration as:

$$w \leftarrow w - H(w)^{-1}g(w) \approx w - E(H(w))^{-1}g(w), \tag{36}$$

where $-E(H(w))$ denotes the expected Hessian, i.e., the Fisher information matrix $I(w)$, which is computed as:

$$\begin{aligned} I(w) = -E(H(w)) = Var(g(w)) &= \frac{1}{\sigma^2}X\,\mathrm{Var}\left((y - \mu_y) \odot \theta^2\right)X^T \\ &= \frac{1}{\sigma^2}X\,\mathrm{diag}\left(Var(y_t)\left(\frac{\partial \theta_t}{\partial \eta_t}\right)^2\right)X^T \\ &= \frac{1}{\sigma^2}X\,\mathrm{diag}\left(\sigma\frac{\partial \mu_y}{\partial \theta_t}\left(\frac{\partial \theta_t}{\partial \eta_t}\right)^2\right)X^T \\ &= \frac{1}{\sigma}X\,\mathrm{diag}\left(Var(y_t)^{-1}\left(\frac{\partial \mu_{y_t}}{\partial \eta_t}\right)^2\right)X^T \\ &= \frac{1}{\sigma}X\,\mathrm{diag}\left(\frac{\partial \theta_t}{\partial \mu_{y_t}}\right)X^T \\ &= \frac{1}{\sigma}X\,\mathrm{diag}(\theta_t^2)X^T. \end{aligned} \tag{37}$$

Given Equation (34) and Equation (37), we have Equation (36) as:

$$\begin{aligned} w &\leftarrow w - E(H(w))^{-1}g(w) \\ &= w + \left(X\,\mathrm{diag}(\theta_t^2)X^T\right)^{-1}X\left((y - \mu_y) \odot \theta^2\right). \end{aligned} \tag{38}$$

This iterative procedure continues until convergence, yielding the maximum likelihood estimates for the parameters w.

Note 10 SIGEL-ETC

As shown in **Main Text, Fig. 6A**, SIGEL-ETC is essentially a GAN model consisting of a generator $\mathcal{G}$ and a discriminator $\mathcal{D}$. Specifically, let $Y \in \mathbb{R}^{N \times H}$ denote the original gene expression matrix from a dataset with full transcriptomic coverage, where N is the number of genes and H is the number of spatial spots within the dataset. The encoder of SIGEL's MAE is denoted as E with parameters W. Then, we have SGRs matrix as $S \in \mathbb{R}^{N \times D} = E(Y, W)$, where D is the SGR dimension. S serves as the inputs to SIGEL-ETC's generator. Let $X \in \mathbb{R}^{\widehat{N} \times M}$ denote the gene expression matrix of the target FISH-based dataset, where $\widehat{N} \ll N$ represents the number of its covered genes, while M represents the number of spatial spots. SIGEL-ETC's generator consists of three subcomponents: an encoder, a decoder, and a memory bank. Explicitly, for a given gene i included in X, the encoder passes its SGR, s_i, through several feed-forward layers to generate a non-linear projection $z_i \in \mathbb{R}^d$. The memory bank is an embedding queue $Q \in \mathbb{R}^{N_{\text{mem}} \times d}$ filled with z, where N_{mem} denotes the number of in-memory *embeddings*. It provides an attention-based means to reconstruct z as $\tilde{z} \in \mathbb{R}^d$:

$$\tilde{\mathbf{z}}_i = \boldsymbol{Q}^T \text{softmax}\left(\frac{\boldsymbol{Q}\mathbf{z}_i}{\tau}\right) \tag{39}$$

where τ is a temperature hyperparameter. $\boldsymbol{Q}$ is continuously updated during training by enqueuing recent ž and dequeuing the oldest. This updating strategy maintains a balance between preserving previously learnt features and adapting to new features to mitigate the mode collapse risk. The decoder is a Multi-Layer Perceptron (MLP) network for regenerating the spatial gene expression vector $\widehat{\boldsymbol{x}}_i$ from $\widetilde{z}_i$. SIGEL-ETC's discriminator $\mathcal{D}$ consists of an MLP-based encoder and a classifier. It is trained to distinguish between $\boldsymbol{x}_i \in X$ and the corresponding $\widehat{\boldsymbol{x}}_i$. The total loss functions for the generator ($\mathcal{L}_G en$) the discriminator ($\mathcal{L}_D$) are defined as:

$$\begin{gathered}
\widehat{\boldsymbol{x}} = \mathcal{G}(s), \\
\mathcal{L}_{Gen} = \alpha \mathcal{L}_{rec} + \beta \mathcal{L}_{adv} = \alpha \mathbb{E}(\|\boldsymbol{x} - \widehat{\boldsymbol{x}}\|_1) - \beta(\mathbb{E}[\boldsymbol{D}(\widehat{\boldsymbol{x}})]), \\
\mathcal{L}_D = \mathbb{E}[\boldsymbol{D}(\widehat{\boldsymbol{x}})] - \mathbb{E}[\boldsymbol{D}(x)] + \lambda \mathbb{E}\left[(\|\nabla \boldsymbol{D}(\xi)\|_2 - 1)^2\right]
\end{gathered} \tag{40}$$

where $\xi = \epsilon\hat{x} + (1-\epsilon)x, \epsilon \in (0, 1)$. Here, $\mathcal{L}_r ec$ denotes the gene reconstruction loss, while $\mathcal{L}_a dv$ the adversarial loss. $\alpha, \beta, and\ \lambda \geq 0$ represent the weights of each loss function. $\mathcal{D}(\widehat{\boldsymbol{x}}_i) \in \boldsymbol{R^h}$ is the discriminator's output for $\widehat{\boldsymbol{x}}_i$, and $\mathbb{E}[\mathcal{D}(\widehat{\boldsymbol{x}}_i)] \in [0, 1]$ represents the probability that $\widehat{\boldsymbol{x}}_i$ is classified as real by D. Additionally, a gradient penalty term on ξ is included in $\mathcal{L}_D$ to ensure the Lipschitz continuity of the discriminator for maintaining the stability of the adversarial training process[41]. The loss of SIGEL-ETC's generator is instrumental in finetuning the weights of the MAE encoder in SIGEL's module I, which enables the SGRs to adapt to the particular semantics inherent in the FISH-based ST dataset. This finetuning facilitates the generation of those genes uncovered in the

FISH-based dataset with optimized fidelity, with its process explicitly describedas:

$$W \leftarrow W - \gamma \frac{\partial \mathbb{E}(Y, W)}{\partial W} \frac{\partial \mathcal{L}_{Gen}}{\partial \mathbb{E}(Y, W)} \tag{41}$$

where γ is the learning rate. Upon training completion, the spatial gene expression profile of any uncovered gene j can be imputed as $\widehat{\boldsymbol{x}}_j = \boldsymbol{\mathcal{G}}(s_j)$.

Note 11 SIGEL-SVG

For each real gene in the ST dataset, we first simulate five spatially homogeneous genes with independently and identically distributed expression profiles that mirror the real gene at each spot. Here, we assume the read counts of a given real gene at every spatial spot follows either a negative binomial (NB) or a zero-inflated negative binomial (ZINB) distribution. The former distribution has four parameters, including the mean μ, the variance υ, the dispersion s, and the probability of a positive event p, while the latter has an additional parameter, the proportion of zero inflation π. These parameters can be estimated from the observed spatial expression vector $x \in R^d$, where d denotes the number of spatial spots. With the estimated parameters, either the "rnegbin" of the R package "MASS" or the "zinb" function of the R package "rzinb" are used to simulate spatially homogeneous genes that follow NB or ZINB distribution, respectively. Gene specific NB parameters are estimated by minimizing the negative log-likelihood of observed gene read counts w.r.t the parameters using PyTorch's autograd[38].The estimation of gene specific ZINB parameters is more involved. Explicitly, we have the following equations among the ZINB parameters:

$$\begin{aligned} v &= \mu + \frac{\mu^2}{s}, \\ \mu &= \frac{s(1-p)}{p}. \end{aligned} \tag{42}$$

The relationships between the ZINB parameters and the observed values of zero ratio r, the sample mean m and the variance σ^2 of x are:

$$\begin{aligned} r &= \pi + (1-\pi)p^s = \frac{\sum_{i=1}^{d} \mathbb{I}(x_i = 0)}{d} \\ m &= (1-\pi)\mu = \frac{\sum_{i=1}^{d} x_i}{d} \\ \sigma^2 &= \pi m^2 + (1-\pi)\mathbb{E}\left(\sum_{i=1}^{d}(x_i - m)^2\right) \\ &= \pi m^2 + (1-\pi)(v + \mu^2 - 2m\mu) \\ &= (1-\pi)v + \pi(1-\pi)\mu^2 = \frac{\sum_{i=1}^{d}(x_i - m)^2}{d} \end{aligned} \tag{43}$$

We design a numerical algorithm to iteratively solve the equations and approximate the values of the ZINB parameters, as elaborated in Note 13 Supplementary Algorithm. Finally, simulated genes are encoded into SGRs. The spatial variability score of any given real gene is calculated as the average scaled cosine dissimilarity between its SGR and those of the corresponding simulated genes. By ranking spatial variability scores in descending order, we can select a predetermined number of top-ranked genes as SVGs.

Note 12 SIGEL-ISC

SIGEL-ISC requires a gene identity matrix $g \in R^{N \times L}$ to indicate genes' identities in functional groups, where N denotes the number of genes and L the number of groups. Such a matrix can be constructed from SIGEL-generated gene groups:

$$G_{i,j} = \begin{cases} 1, if \ I(i) = j \\ 0, otherwise \end{cases} \tag{44}$$

where $I(i)$ is a function indicating gene i's group identity. Alternatively, G can be constructed from a gene-gene similarity matrix, $S \in R^{N \times N}$,calculated as the normalized Gram matrix of SGRs:

$$S = Diag(|Z|)^{-1} ZZ^T Diag(|Z|)^{-1} \tag{45}$$

where $Z \in R^{N \times N}$ denotes the SGR matrix, N the number of genes, and D the SGR dimension. is then converted to degree-normalized mutual -nearest neighbors adjacency matrix $\widetilde{\mathcal{A}} \in \mathbb{R}^{N \times N}$:

$$\mathcal{A}_{i,j} = \begin{cases} 0, & \text{if } i = j, \\ 1, & \text{if } i \neq j, \ i \in N_j(k), \ j \in N_i(k), \\ 0, & \text{otherwise.} \end{cases}$$

$$\widetilde{\mathcal{A}} = \Delta^{-1} \mathcal{A} \tag{46}$$

where $N_i(k)$ denote the k-nearest neighbors of gene i on $S.\Delta$ is the diagonal degree matrix of $\mathcal{A}_i$ with $\Delta_{i,i} = \sum_j .\mathcal{A}_{i,j}$. Shi-Malik spectral clustering[42] is performed on $\widetilde{\mathcal{A}}$ to get G. SIGEL-SVG is then utilized to acquire gene spatial variability scores $\mathcal{V} \in R^N$, based on which a redundancy filtering matrix $\mathcal{F} \in R^{N \times N}$ is constructed:

$$\mathcal{F}_{i,i} = \begin{cases} 1, if \ R(\mathcal{V}_i) \leq \sigma \\ 0, otherwise \end{cases} \tag{47}$$

where $R(\mathcal{V}_i)$ is a function that returns the ranking percentile of $\mathcal{V}_i$ in $\mathcal{V}, \sigma$ is a pre-specified threshold for controlling redundancy. Finally, $\mathcal{F}$ is applied to the original spatial gene expression matrix $X \in R^{M \times N}$, where M denotes the number of spatial spots:

$$\widetilde{X} = X\mathcal{F}. \tag{48}$$

Note that $\widetilde{X}$ represents an informational-efficient version of X and are subsequently fed into a two-layer convolutional graph neural network to generate spot embeddings, on which clustering is performed.

Note 13 Supplementary Algorithm

Input: target gene's expression vector: $\mathbf{x} \in \mathbb{R}^d$; max iteration: MaxIter; stopping threshold: δ;

Output: π, μ, v, s, p;

1. $r = \frac{\sum_{i=1}^{d} \mathbb{I}(x_i=0)}{d}$, $m = \frac{\sum_{i=1}^{d} x_i}{d}$, $\sigma^2 = \frac{\sum_{i=1}^{d} (x_i-m)^2}{d}$

2. $\pi_0 = \alpha \cdot r$, $\alpha \in (0,1)$. Due to the sparsity of the gene count matrix, α is set to be 0.99 in our case.

3. **for** $j \in [0, \mathrm{MaxIter}]$ **do**

   (a) $\mu_j = \frac{m}{1-\pi_j}$

   (b) $v_j = \max\left(\frac{\sigma^2-\pi_j(1-\pi_j)\mu_j^2}{1-\pi_j}, \text{1e-5}\right)$

   (c) $s_j = \max\left(\frac{\mu_j^2}{v_j-\mu_j}, 1\right)$

   (d) $p_j = \max\left(\frac{s_j}{\mu_j+s_j}, \text{1e-6}\right)$

   (e) $\pi_{j+1} = \frac{r-p_j^{s_j}}{1-p_j^{s_j}}$

   (f) **if** $|\pi_j - \pi_{j+1}| \leq \delta$, **then Stop**

4. **end for**

5. **return** $\pi = \pi_{j+1}$; $\mu = \mu_j$; $v = v_j$; $s = s_j$; $p = p_j$
